# Discovery of a brain penetrant small molecule antagonist targeting LPA1 receptors to reduce neuroinflammation and promote remyelination in multiple sclerosis

**DOI:** 10.1038/s41598-024-61369-9

**Published:** 2024-05-08

**Authors:** Michael M. Poon, Kym I. Lorrain, Karin J. Stebbins, Geraldine C. Edu, Alexander R. Broadhead, Ariana O. Lorenzana, Bryanna E. Paulson, Christopher S. Baccei, Jeffrey R. Roppe, Thomas O. Schrader, Lino J. Valdez, Yifeng Xiong, Austin C. Chen, Daniel S. Lorrain

**Affiliations:** Contineum Therapeutics, San Diego, CA 92121 USA

**Keywords:** Oligodendrocyte, Multiple sclerosis

## Abstract

Multiple sclerosis (MS) is a chronic neurological disease characterized by inflammatory demyelination that disrupts neuronal transmission resulting in neurodegeneration progressive disability. While current treatments focus on immunosuppression to limit inflammation and further myelin loss, no approved therapies effectively promote remyelination to mitigate the progressive disability associated with chronic demyelination. Lysophosphatidic acid (LPA) is a pro-inflammatory lipid that is upregulated in MS patient plasma and cerebrospinal fluid (CSF). LPA activates the LPA1 receptor, resulting in elevated CNS cytokine and chemokine levels, infiltration of immune cells, and microglial/astrocyte activation. This results in a neuroinflammatory response leading to demyelination and suppressed remyelination. A medicinal chemistry effort identified PIPE-791, an oral, brain-penetrant, LPA1 antagonist. PIPE-791 was characterized in vitro and in vivo and was found to be a potent, selective LPA1 antagonist with slow receptor off-rate kinetics. In vitro, PIPE-791 induced OPC differentiation and promoted remyelination following a demyelinating insult. PIPE-791 further mitigated the macrophage-mediated inhibition of OPC differentiation and inhibited microglial and fibroblast activation. In vivo, the compound readily crossed the blood–brain barrier and blocked LPA1 in the CNS after oral dosing. Direct dosing of PIPE-791 in vivo increased oligodendrocyte number, and in the mouse experimental autoimmune encephalomyelitis (EAE) model of MS, we observed that PIPE-791 promoted myelination, reduced neuroinflammation, and restored visual evoked potential latencies (VEP). These findings support targeting LPA1 for remyelination and encourage development of PIPE-791 for treating MS patients with advantages not seen with current immunosuppressive disease modifying therapies.

## Introduction

Multiple sclerosis (MS) is a chronic, progressive disease characterized by inflammatory demyelination. This results in disruption of neuronal transmission and ultimately, neurodegeneration. While current immunomodulatory therapies limit inflammation and further demyelination, they do not deliberately address remyelination and are consequently limited in their ability to impact disease progression. As a result, identification of mechanisms that positively impact remyelination has been an area of intense interest.

Lysophosphatidic acid (LPA), a well-known signaling phospholipid, is elevated in the cerebrospinal fluid (CSF) and serum of patients with MS compared to non-inflammatory, non-vascular neurological disease patient samples. This is likely due to infiltration of LPA from the periphery as well as an overexpression of autotaxin in the CNS, the enzyme primarily responsible for LPA synthesis, in MS patients^1–4^. Aberrant levels of LPA in the central nervous system (CNS) may, in turn, promote local neuroinflammation, myelin loss, and limit remyelination through the activation of specific receptors such as the LPA1 receptor.

LPA1 is expressed in oligodendroglial lineage cells. In vitro, oligodendrocyte precursor cells (OPCs) from LPA1-null mice show an enhanced capacity to differentiate into mature oligodendrocytes^5^. Further, LPA1-null mice show significantly improved clinical scores in the myelin oligodendrocyte glycoprotein-induced experimental autoimmune encephalitis (MOG-EAE) model^6^. LPA1 blockade also alleviates inflammation and fibrosis, both of which are contributing factors to MS^7–9^. These data combine to suggest that an LPA1 antagonist may enable a multipronged approach towards disease treatment, and that the identification of a potent, selective, brain penetrant LPA1 antagonist could make a significant impact on the treatment of inflammatory demyelinating diseases such as MS.

We have identified and profiled PIPE-791, a potent, small molecule, brain-penetrant, LPA1 antagonist. Using a combination of in vitro and in vivo models, we show that PIPE-791 induces OPC differentiation into oligodendrocytes and demonstrates efficacy in the MOG-EAE mouse model of MS. We also show that LPA1 may impact other MS-related mechanisms beyond OPC differentiation, including inflammation and fibrosis. Careful pharmacokinetic characterization of PIPE-791 also revealed a slow binding kinetic that resulted in unusually long CNS receptor occupancy. Together, these results encourage the further development of PIPE-791 as a treatment for multiple sclerosis.

## Results

### PIPE-791 is a potent, small molecule LPA1 antagonist

Though several LPA1 antagonists have been optimized for peripheral indications, none have demonstrated adequate brain penetration for CNS studies or indications. As such, we developed PIPE-791, a brain-penetrant, orally bioavailable, small molecule LPA1 antagonist.

We first tested PIPE-791 in a competitive membrane filter binding assay using membranes from cells overexpressing human LPA1. Several concentrations of PIPE-791 were co-incubated with these membranes in the presence of an LPA1 selective radioligand and then filtered^10^. We found that PIPE-791 bound human LPA1 with high affinity with a calculated K_i_ of 0.752 nM (IC_50_ 2.63nM, Table 1).

**Table 1:** Summary table of PIPE-791 in vitro radioligand binding selectivity profile in Ca^+2^ mobilization.

Properties	In vitro profile
Radioligand binding Ki (nM)	0.752 (IC_50_: 2.63)
Functional LPA1 Ca^+2^ mobilization (nM, 30 m)	91.8
Functional LPA1 Ca^+2^ mobilization (nM, 24 h)	9.9
Fold selectivity over LPA2^a^	35.4×
Fold selectivity over LPA3	35×

^a^Selectivity assessed using a 3 h incubation of PIPE-791.

**Figure 1:**
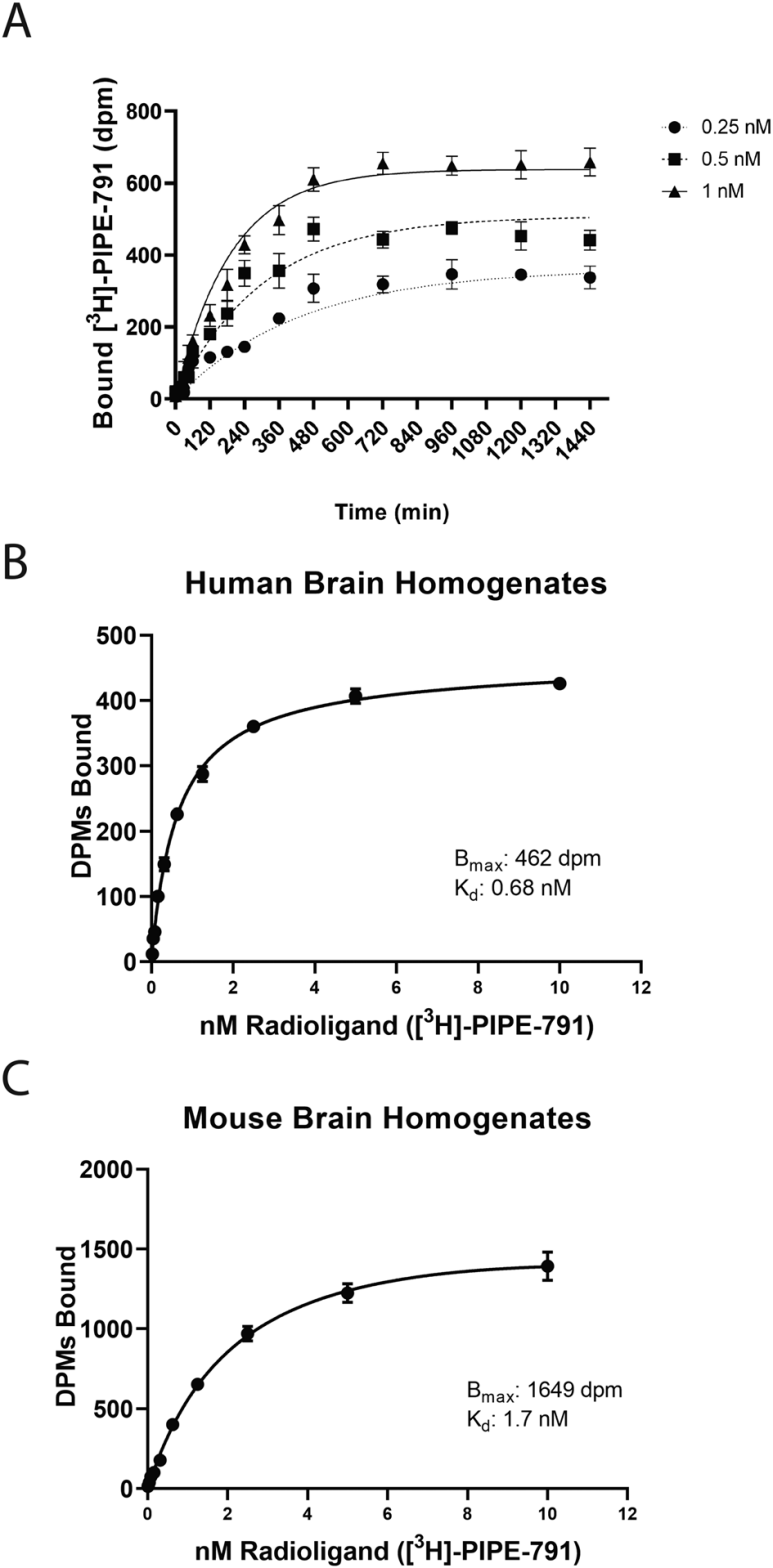
Pharmacological profile of PIPE-791. (**A**) Binding kinetics of PIPE-791 in recombinant membranes. Three concentrations of 3H-PIPE-791 (0.25, 0.5 and 1 nM) were incubated with 1 µM PIPE-791 then added to membranes at different time points, resulting in a calculated t_1/2_ of 519 min (graphs are mean ± SD, n = 4). (**B**,**C**) Saturation binding was performed using increasing concentrations of [^3^H]-PIPE-791 in mouse or human brain homogenate (mean ± SD, n = 4).

Next, we examined the kinetics of PIPE-791 binding to human LPA1 receptor (hLPA1) in a recombinant membrane setting. We used a forward kinetic method where 1 µM PIPE-791 was mixed with 0.25, 0.5, or 1 nM of [^3^H]-PIPE-791. Binding was initiated by adding hLPA1 overexpressing membranes at different time points (ranging from 1 min to 24 h) and filtered. We found that PIPE-791 exhibited slow kinetics with a calculated t_1/2_ of 8.65 h (Fig. 1A, Table 2).

**Table 2:** Summary of PIPE-791 kinetic data. Values were derived using 3 concentrations of [^3^H]-PIPE-791.

Parameter	Value
K_d_	3.41 × 10^–10^ M
K_off_	0.001334 min^−1^
K_on_	3,912,258 M^−1^ min^−1^
t_1/2_	519 min
[^3^H]-PIPE-791 conc	0.25, 0.5, 1 nM

The binding affinity of PIPE-791 to the LPA1 receptor was also assessed in a native tissue setting by performing saturation binding experiments in mouse and human brain homogenates. Using [^3^H]-PIPE-791, saturation binding in human brain tissue revealed a K_d_ of 0.68 nM and a B_max_ of 462 dpm (disintegrations per minute, Fig. 1B). In mouse brain tissue, we observed a K_d_ of 1.7 nM and a B_max_ of 1649 dpm (Fig. 1C). The similarity between these two species suggests that findings in the mouse brain should be translatable to human.

To assess selectivity, PIPE-791 was tested in a functional calcium mobilization assay in cells overexpressing hLPA1 and using either a 30 min or 24 h pre-incubation period prior to LPA addition (Table 1). The slow on-rate kinetics of PIPE-791 likely contribute to the potency shift observed between the 30 min and the 24 h calcium mobilization assay. PIPE-791 also showed selectivity against the two most homologous LPA receptor isoforms, LPA2 (35.4-fold) and LPA3 (35-fold) (Table 1). We also evaluated PIPE-791 in a Eurofins off-target panel (SAFETYscan E/IC50 ELECT) and observed no appreciable activities at a test concentration of 30 µM (Supplementary Fig. 1).

### In vivo pharmacokinetics and binding kinetics

Using in vitro methods, we have shown that PIPE-791 exhibits unusually slow association kinetics. We wanted to see if this property could be recapitulated in vivo, particularly in the context of plasma concentration and brain LPA1 receptor occupancy across time.

In the first experiment, mice were dosed intravenously with [^3^H]-PIPE-791 and brains harvested at different time points. In agreement with the kinetics observed in vitro, receptor binding of [^3^H]-PIPE-791 in the brain gradually increased over time, plateauing between 24 and 72 h. Receptor bound radioligand levels then declined by 7 days, demonstrating that binding was reversible. Specific binding was determined at the 2 and 24 h timepoints by competition with unlabeled PIPE-791 that was administered orally at a dose of 3 mg/kg (Supplementary Fig. 2).

**Figure 2:**
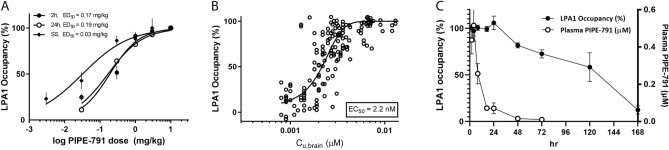
PIPE-791 has distinct in vivo brain occupancy kinetics. (**A**) Dose-occupancy was assessed 2 h or 24 h after administration of PIPE-791. For the steady state condition, mice were dosed once daily for 4 days then occupancy assessed 2 h after the fourth dose (mean ± SEM, n = 6). (**B**) Pooled analysis of unbound brain concentration (C_u_, _brain_) plotted versus occupancy. (**C**) Time course of 3 mg/kg PIPE-791 plotted against brain receptor occupancy (left y-axis) and plasma concentration (right y-axis) highlighting its extended brain receptor occupancy and disconnect with plasma concentration (mean ± SEM, n = 6).

The in vivo receptor occupancy of PIPE-791 was characterized using a novel, selective, and brain-penetrant LPA1 radioligand ([^3^H]-OPC3497) (Supplementary Fig. 3). This radioligand displays relatively fast on and off kinetics making it more suitable for in vivo occupancy studies. Following a single oral dose, PIPE-791 inhibited [^3^H]-OPC3497 binding to mouse brain in a dose-dependent manner with an ED_50_ of 0.17 mg/kg and 0.19 mg/kg when administered 2 h or 24 h prior to radioligand, respectively (Fig. 2A). The unbound brain EC_50_ (C_u, brain_) was 2.2 nM (Fig. 2B).

When PIPE-791 was administered once daily for 4 consecutive days to reach steady state, the LPA1 receptor occupancy ED_50_ decreased to 0.03 mg/kg while the unbound brain EC_50_ remained relatively unchanged at 2.4 nM. The shift in ED_50_ following repeat dosing is consistent with the slow receptor binding kinetics associated with PIPE-791 (Fig. 1A).

To further explore the pharmacokinetic/pharmacodynamic (PK/PD) relationship, mice were dosed orally with 3 mg/kg of PIPE-791 and its plasma concentration and the corresponding brain receptor occupancy were evaluated out to 7 days. LPA1 occupancy was sustained for 24 h and slowly decreased out to 7 days. Plasma concentrations, on the other hand, peaked early (2 h) and decreased rapidly by 24 h and were below the limit of detection after 3 days. This disconnect in the PK/PD relationship is again consistent with the slow receptor off-rate observed with PIPE-791 (Fig. 2C).

### LPA1 is expressed in oligodendrocyte precursor cells

**Figure 3:**
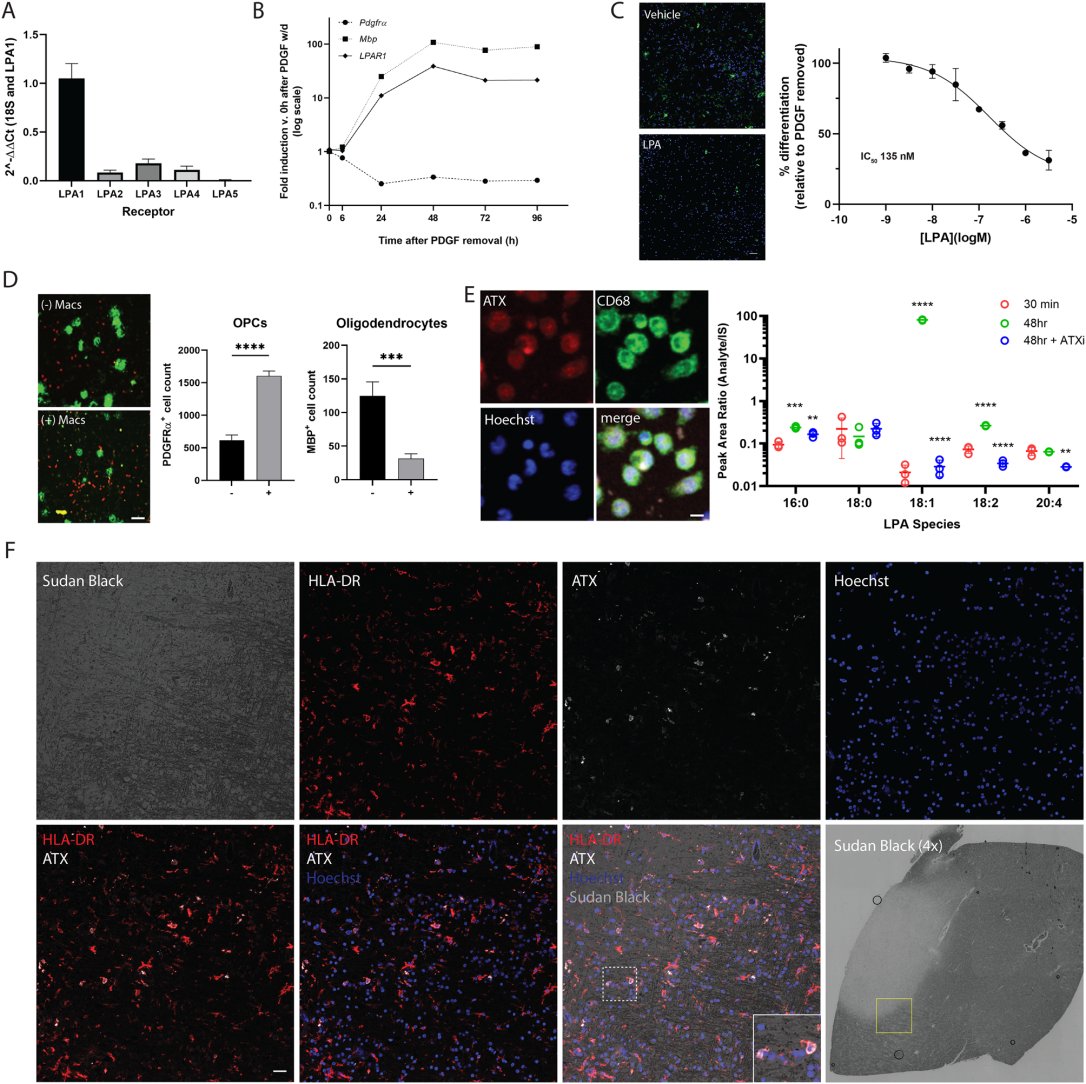
LPA1 is expressed on OPCs. (**A**) Quantitative PCR using primers against the *Lpar1* through *5* in rat O4^+^ OPCs. Error bars are SEM, n = 3. (**B**) LPA1 expression over the course of differentiation in response to PDGF withdrawal. (**C**) Exogenous addition of LPA suppresses rat OPC differentiation upon PDGF withdrawal. Left, representative images of OPC cultures after PDGF withdrawal in the presence of LPA; top: no LPA, bottom LPA (1 µM), MBP (green) counterstained with Hoechst (blue). Scale bar 50 µm. Right, graph of LPA dose responsive suppression of OPC differentiation by LPA, IC_50_ 135 nM. (**D**) Macrophages express autotaxin and release LPA. OPCs were plated in the upper compartment of a Transwell culture plate in the presence (+) or absence (−) of physically separated macrophages (bottom compartment). After PDGF withdrawal, fewer MBP^+^ oligodendrocytes (and more PDGFRα^+^ OPCs) were observed in the macrophage co-culture condition, (MBP p = 0.0008; PDGFRα p < 0.0001, mean ± SEM, n = 8, t-test). Representative images (left) of oligodendrocytes (MBP, green) and OPCs (PDGFRα, red) in the absence (− Macs) or presence of macrophages (+ Macs). Scale bar 50 µm. (**E**) Left, representative images of macrophages immunostained using antibodies against the LPA synthetic enzyme, autotaxin ATX (red), the macrophage marker CD68 (green). Cells were counterstained with Hoechst (blue). Scale bar: 5 µm. Right, quantification of LPA species in macrophage-conditioned media. Media LPA levels increase from 30 m to 48 h after plating. 1 µM PF-8380 (ATX inhibitor) prevents the induction of several LPA species at 48 h, including 18:1 (one way ANOVA, Tukey’s posthoc, significance against 48 h/no inhibitor group, **p < 0.01, *** < 0.001, ****p < 0.0001). (**F**) Tissue from an MS patient was stained with antibodies against HLA-DR (red), autotaxin (white) and counterstained with Hoechst (blue) and the myelin dye, Sudan Black. Scale bar: 25 µm. Inset (white box) is a magnified view showing HLA-DR^+^/ATX^+^/Hoechst^+^ cells. Bottom right is 4 × Sudan black image of section from where images were acquired. Magnified region of interest is highlighted in yellow. Myelin-poor lesion is lighter area where little staining is observed.

Having identified and characterized PIPE-791 as an LPA1 selective antagonist in both in vitro and in vivo contexts, we sought to better understand LPA1 expression CNS, particularly in the context of multiple sclerosis. An earlier report had described the expression of LPA1 in OPCs^11^. We have since independently confirmed these data by examining *Lpar1* expression in OPCs enriched using the OPC-specific surface marker O4. O4^+^ OPCs were isolated from rat cortex and LPA1-5 receptors were assayed by quantitative PCR. In addition to confirming the presence of *Lpar1*, we observed that *Lpar2*–*5* mRNA expression was significantly lower in OPCs (Fig. 3A).

We then *examined Lpar1* expression as OPCs differentiated into oligodendrocytes. Using platelet-derived growth factor (PDGF) withdrawal to induce OPC differentiation, we examined *Lpar1* expression at several timepoints^12^. Upon PDGF removal, we observed an increase in the oligodendrocyte marker, myelin basic protein (*Mbp*) and a concurrent decrease in the OPC marker *Pdgfra*, thereby confirming differentiation. Interestingly, over the course of differentiation, we also observed an increase *in Lpar1* expression, consistent with literature and databases showing that mature oligodendrocytes also express *Lpar1* (Fig. 3B).

Given the expression *of Lpar1* on OPCs, we wanted to know how direct, exogenous application of LPA would affect OPC differentiation. Using the same PDGF withdrawal paradigm to induce OPC differentiation, we added various concentrations of LPA to the culture media and quantified MBP^+^ oligodendrocytes 3 days later. In doing so, we observed a dose dependent inhibition of OPC differentiation in response to LPA treatment (Fig. 3C) thereby suggesting LPA receptor activation by LPA as a negative regulator of this process. Although these results alone do not directly implicate LPA1, cultured OPCs from LPA1 knockout mice also show enhanced OPC differentiation compared to wildtype controls ^5^.

### Macrophage-secreted factors suppress OPC differentiation

During MS, inflammatory macrophages invade the CNS, destroying the myelin sheath and releasing factors that prevent subsequent myelin repair^13^. While macrophage-oligodendrocyte co-culture systems have been used to show that macrophages phagocytose oligodendrocytes, such cultures have not been used to study the secretion of diffusible factors^14–16^.

To better understand the impact of macrophage-secreted factors on OPCs, we utilized a Transwell culture system to spatially separate macrophages and OPCs but still allow for factor exchange. Rat peritoneal macrophages were plated in the bottom compartment while rat OPCs were plated on a Transwell insert above the macrophages. Upon PDGF withdrawal, we observed an overall suppression of OPC differentiation; specifically, a decrease in MBP^+^ oligodendrocytes and an increase in PDGFRα^+^ OPCs, when OPCs were cultured in the presence of macrophages (Fig. 3D).

Autotaxin (ATX), an enzyme responsible for LPA synthesis, is elevated in CSF samples from multiple sclerosis patients and may be an underlying source of elevated LPA levels in the CNS during disease. By immunocytochemistry, we confirmed that the CD68^+^ macrophages express autotaxin (ATX), suggesting that LPA may be one of possibly several secreted, diffusible factors involved in suppressing OPC differentiation (Fig. 3E)^6,17^. To confirm the release of LPA from these macrophages, macrophage-conditioned media was taken 30 min or 48 h after plating and various LPA species were measured by mass spectrometry^18^. We observed a significant increase in several LPA species over time. Most notably, 18:1 LPA which has been implicated in LPA-mediated disorders such as neuropathic pain, showed the most significant increase over time^19^. Importantly, the increase in LPA was also inhibited in the presence of the autotaxin inhibitor, PF-8380 (1 µM, Fig. 3E). Together, these data build on previous observations that in addition to destroying oligodendrocytes, macrophages may also limit myelin repair by suppressing OPC differentiation through the release of diffusible factors such as LPA.

### Autotaxin expressing cells are found near lesions in MS patient brain tissue

To see whether LPA may be involved in MS, we obtained human MS tissue and examined autotaxin expression around the lesion border. Fresh frozen MS tissue was stained with Sudan Black to demarcate demyelinated regions^20^. The tissue was immunostained for ATX and HLA-DR (a marker for inflammatory microglia and macrophages,^21^). At the lesion, HLA-DR^+^ cells were abundant near the lesion border. Importantly, there was substantial autotaxin colocalization with HLA-DR^+^ cells suggesting that cells such as microglia or macrophages may act during multiple sclerosis to increase local levels of LPA at the lesion sites. (Fig. 3F and Supplementary Fig. 4).

### PIPE-791 induces OPC differentiation in dissociated culture and can overcome inhibition by macrophage-released factors

**Figure 4:**
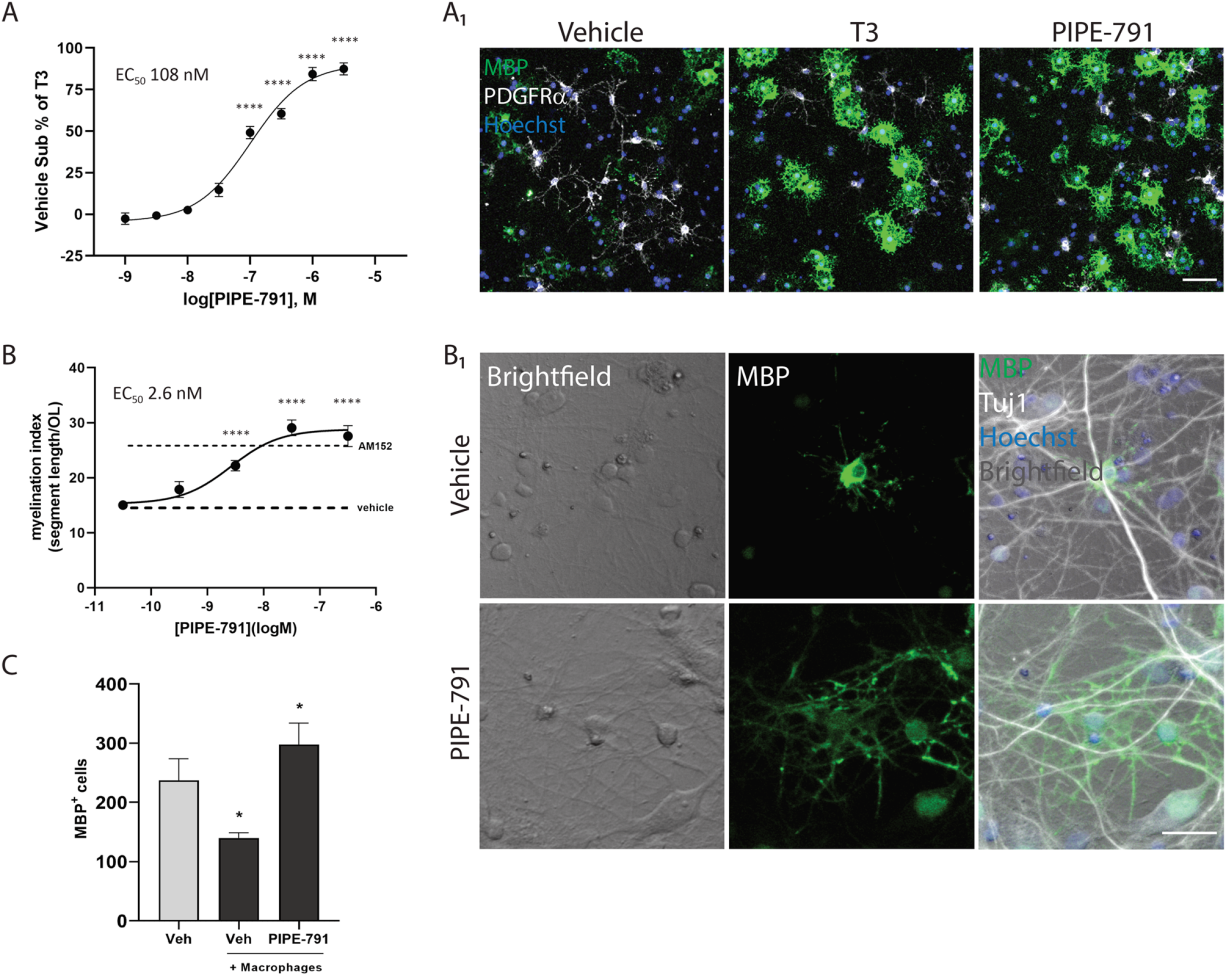
PIPE-791 induces OPC differentiation. (**A**) Dissociated rat oligodendrocyte precursor cells were treated with PIPE-791 and immunostained with an antibody against MBP (green) and the OPC marker PDGFRα (white). A dose dependent increase in MBP^+^ oligodendrocytes was observed. Graphs plotted as vehicle subtracted percent of T3 induced differentiation (EC_50_ 108 nM, mean ± SEM, n = 6). Values were compared by ANOVA with Dunnet’s vs vehicle: ****p < 0.0001; T3 was p < 0.0001 by t-test to vehicle). A_1_, representative image at 1 µM PIPE-791. Scale bar: 50 µm. (**B**) PIPE-791 induces oligodendrocytes that can myelinate axons in a rat cortical myelination assay. Cells were immunostained against MBP (green), Tuj1 (white), and counterstained with Hoechst (blue). Linear myelin segments were measured to obtain a myelination index, resulting in an EC_50_ of 2.6 nM. 1 µM AM152 was used as a positive control, mean ± SEM, n = 4. Values were compared by ANOVA with Dunnett’s vs vehicle, ****p < 0.0001; AM152 was p < 0.0001. B_1_, representative image at 300 nM PIPE-791. Scale bar: 25 µm. (**C**) PIPE-791 (300 nM) increases MBP^+^ rat oligodendrocytes despite the presence of inhibitory macrophages (mean ± SEM, t-test vs vehicle no macrophages, n = 8).

Having characterized PIPE-791 as a potent antagonist of LPA1, we determined whether the treatment of OPCs with PIPE-791 could induce their differentiation. Primary rat OPCs were isolated and cultured in the presence of PDGF. One day later, PDGF was removed and various doses of PIPE-791 were added to the cultures. These cultures were fixed 3 days post-PIPE-791 treatment and immunostained for MBP. We observed a dose dependent increase in the number of differentiated oligodendrocytes with an EC_50_ of 108 nM (Fig. 4A).

We next tested whether PIPE-791 induced oligodendrocytes could functionally myelinate axons using a rat primary cortical myelination assay described previously^22^. In this assay, cells from embryonic day 15 rat cortex were plated. Over time, axons in the culture are myelinated by oligodendrocytes and myelin segments can be measured. We observed robust myelination following PIPE-791 treatment, with an EC_50_ of 2.6 nM (Fig. 4B). AM152 (BMS-986020), a clinical stage, peripherally-restricted LPA1 antagonist, was used as a reference control.

We then revisited the macrophage-OPC Transwell co-culture to assess whether PIPE-791 could derepress differentiation inhibition by macrophage-secreted factors. Following PDGF withdrawal, we observed a 41% decrease in the number of MBP^+^ oligodendrocytes when co-cultured with macrophages. In the presence of macrophages, addition of PIPE-791 resulted in a 2.1-fold increase in differentiation versus vehicle treated cultures (Fig. 4C).

### PIPE-791 induces OPC differentiation in vivo

To evaluate whether PIPE-791 could increase oligodendrocyte number in vivo, mice were dosed once with 3 mg/kg PIPE-791 and 5 days later, brains collected, and tissue sectioned. Sections were immunostained for the mature oligodendrocyte marker, CC1, and the oligodendroglial marker, OLIG2 to represent the total OPC/oligodendrocyte population. In doing so, we observed a significant 163% increase in CC1^+^/OLIG2^+^ oligodendrocytes suggesting OPC differentiation (21).

### PIPE-791 induces OPC differentiation in human slice culture

**Figure 5:**
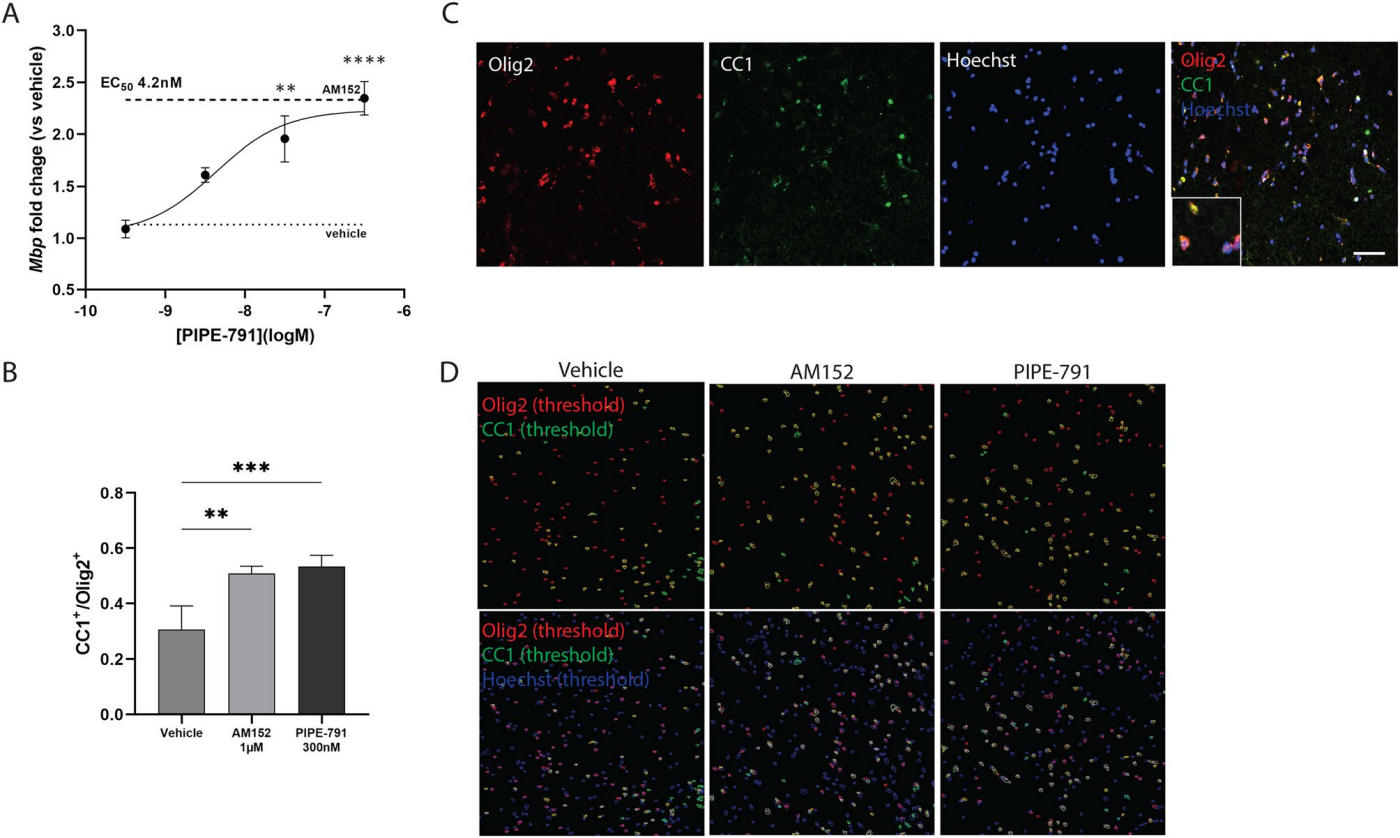
PIPE-791 induces oligodendrocytes in human cortical slice culture. (**A**) Cortical slices from fresh donor human brain were generated and treated with various concentrations of PIPE-791. *Mbp* transcript was quantified by qPCR resulting in an EC_50_ of 4.2 nM (mean ± SEM, n = 8; values were compared using ANOVA and Dunnett’s versus vehicle, **p < 0.01, ****p < 0.0001). (**B**) Human cortical slices treated with vehicle, 1 µM AM152, or 100 nM PIPE-791 were also processed by immunohistochemistry using antibodies against the mature oligodendrocyte marker, CC1 and the oligodendroglial marker Olig2. A significant increase in CC1^+^ cells was observed, comparable to the positive control, AM152 (**p = 0.0016, ***P = 0.0007, mean ± SEM, n = 4 for each group, ANOVA with Tukey’s). (**C**) Representative image of slice stained with, Olig2 (red), CC1 (green), Hoechst (blue). Scale bar 50 µm). Inset is a magnified image of cells co-expressing Olig2, CC1 and Hoechst. (**D**) Thresholded images used for analysis. Olig2^+^ cells are represented as red outlines, CC1 as green, and Hoechst as blue. Only CC1^+^/Olig2^+^/Hoechst^+^ were counted as oligodendrocytes.

PIPE-791 potently antagonizes the human LPA1 receptor and triggers the differentiation of mouse and rat OPCs into functional oligodendrocytes. To assess whether PIPE-791 could similarly induce OPC differentiation in a human context, we evaluated oligodendrocyte markers in human cortical slice cultures after treatment. Cortical slices from regions containing both white and gray matter were generated from fresh human adult donor tissue and cultured for 10 days. PIPE-791 was then added to the culture for 9 days. Analyzing *Mbp* by qPCR showed a dose-dependent increase with an EC_50_ of 4.2 nM (Fig. 5A).

We also used an immunohistochemical endpoint and quantified the number of CC1^+^ cells present after PIPE-791 treatment. At 300 nM, we observed significant differentiation following PIPE-791 treatment. The magnitude of the effect was also comparable to 1 µM AM152. In light, these data suggest that PIPE-791 has the potential to induce OPC differentiation in a human setting (Fig. 5B–D).

### PIPE-791 inhibits microglial activation but does not inhibit phagocytosis

LPA1 has been implicated in the activation of microglia in several disease models, including MOG-EAE^6,24, 25^. In light, we wanted to test whether treating microglia with PIPE-791 could inhibit their activation.

**Figure 6:**
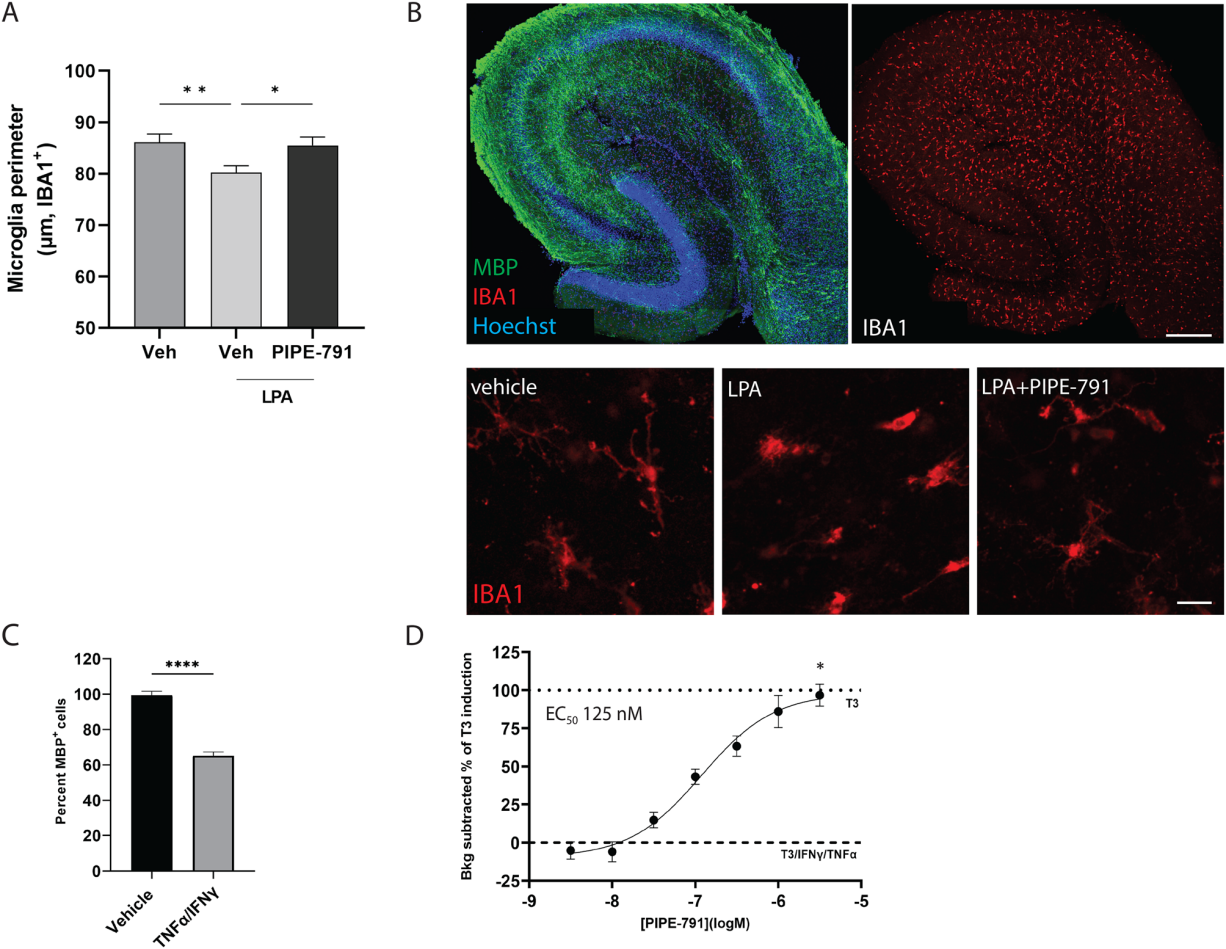
PIPE-791 inhibits microglial activation. (**A**) Mouse hippocampal slices (postnatal day 21) were generated and treated with PIPE-791. LPA was then added to the slices to induce microglial activation. Slices were fixed and stained with an antibody against IBA1 and counterstained with Hoechst. Treatment with LPA converts microglia from a branched, ramified morphology to round and activated and is prevented in the presence of 3 µM PIPE-791. Activation was quantified using cell perimeter length of IBA1^+^/Hoechst^+^ cells (mean ± SEM, n = 4 slices, 3 animals) ANOVA with Tukey’s). Scale bar: 100 µm. (**B**) Example image from slices treated with 10 µM LPA and LPA with PIPE-791. Scale bar: 25 µm. (**C**) Rat OPCs were differentiated with T3 into oligodendrocytes then treated with TNFα/IFNγ resulting a 35% decrease in viability (mean ± SEM, n = 2, 6 wells/n). (**D**) Addition of PIPE-791 prevented oligodendrocyte death in a dose-responsive manner (EC_50_ 125 nM, mean ± SEM, 6 wells/n, graphs are background subtracted to TNFα/IFNγ and plotted as % of T3/vehicle. Values were compared by ANOVA with Dunnett’s vs vehicle, * p < 0.05).

Hippocampal slices from postnatal day 20 mice were generated and transferred into basal media containing PIPE-791 or AM152 for 2 h. Microglial activation was then triggered by the addition of 10 µM LPA, slices incubated for 3 additional hours, then fixed. Slices were immunostained against IBA1 and microglial perimeters were measured as a mean of differentiating between ramified/resting versus rounded/activated microglia. After LPA treatment, we saw a significant increase in the number of rounded IBA1^+^ cells as measured by a decrease in cell perimeter length, indicative of activation^26^. This decrease in perimeter length was inhibited in slices that were treated with LPA in the presence of PIPE-791 or AM152 (Fig. 6A,B and Supplementary Fig. 6).

To see if PIPE-791 impacted the ability of microglia to phagocytose myelin debris, microglia were isolated and cultured in an activated state using complete serum media which inherently contains high levels of LPA^27^. At this point, microglia already displayed an activated, amoeboid morphology. Microglia were then treated with various concentrations of PIPE-791. Fluoromyelin-labeled myelin debris was added for 90 min and then the cultures were fixed. IBA1^+^/fluoromyelin^+^ cells were quantified and normalized to total IBA1^+^ cells. Compared to vehicle, no decrease in myelin phagocytosis was observed at any concentration of PIPE-791 (^28^).

### PIPE-791 reduces meningeal fibroblast activation

Recent work from Dorrier et al., has implicated persistent fibrotic scarring in the CNS in the mouse EAE model and that the contributing cells were of meningeal origin. As LPA and LPA1 have been implicated in the induction and progression of fibrosis, we wanted to see whether PIPE-791 could inhibit the activation of brain meningeal fibroblasts^8,9^. We cultured primary rat or human brain meningeal fibroblasts and applied 10 µM LPA to induce activation. Upon LPA stimulation, we saw a change in morphology that could be quantified as a change in collagen I (COL1^+^) area. Preincubation with PIPE-791 inhibited the increase in COL1^+^ with an IC_50_ of 31.8 nM in rat and 4.5 nM in human meningeal fibroblasts (Supplementary Fig. 8).

### PIPE-791 protects oligodendrocytes from cytokine insult

TNFα and IFNγ are two cytokines that, while not normally expressed in the CNS, are elevated in MS^29^. These cytokines can be secreted by both macrophages and microglia^30,31^. Receptors for these cytokines (TNFR1, TNFR2, and IFNGR) are expressed in oligodendrocytes. Of note, TNFR1 and 2 have been found on oligodendrocytes bordering MS lesions^23,32, 33^. Further, although receptor expression for these cytokines on OPCs is unknown, addition of either of these cytokines impact differentiation in isolated OPC cultures^28,34^. Cultured rat OPCs were differentiated with T3 for 24 h then treated with both TNFα and IFNγ Upon cytokine addition, we observed significant cell death consistent with previous observations (Fig. 6C, left^22,35–37^). To test whether PIPE-791 could afford protection after cytokine insult, cells were treated with TNFα and IFNγ along with various concentrations of compound. In the presence of PIPE-791, we observed dose-dependent protection of MBP^+^ oligodendrocytes with an EC_50_ of 125 nM (Fig. 6D, right). Concurrent assessment of total cellular viability with Alamar blue yielded an EC_50_ of 39.6 nM (data not shown). While the mechanisms underlying this observation remain unknown, the results do suggest PIPE-791 promotes oligodendrocyte survival in addition to differentiation in the context of an inflammatory microenvironment.

### PIPE-791 induces oligodendrocytes in lysolecithin treated mouse organotypic slice cultures

**Figure 7:**
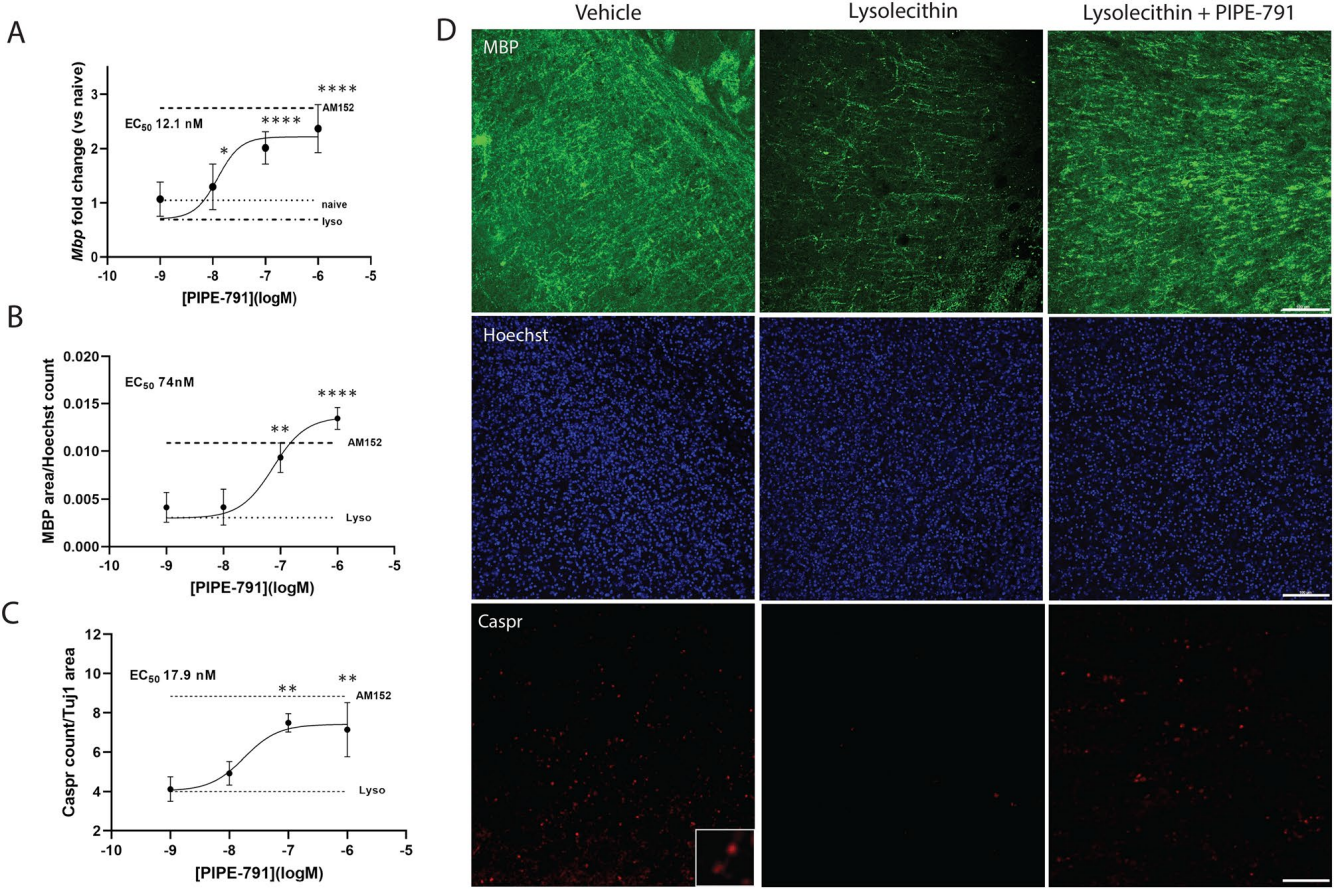
(**A**) Mouse cortical slice cultures were collected at postnatal day 17, cultured for 72 h then treated with lysolecithin for 18 h. Following insult, lysolecithin was removed and treatments added for 3 days. PIPE-791 induced *Mbp* transcript with an EC_50_ of 14.7 nM with efficacy comparable to 1 µM AM152 (mean ± SD, n = 4). (**B**) Mouse slices were prepared as in (**A**) but treated with PIPE-791 for 5 days. Slices were immunostained for MBP (green), Caspr (red), and Hoechst (blue). Images were from regions adjacent to the superior edge of the corpus callosum. A dose dependent increase in the amount of MBP (**B**, EC_50_ 74 nM, naïve value 0.013) and the number of Caspr puncta (**C**, EC_50_ 17.9 nM, naïve value = 12.33) were observed (mean ± SD, n = 4, values were compared using ANOVA and Dunnett’s versus lysolecithin, **p < 0.01, ****p < 0.0001). (**D**) Representative images of MBP (green), Hoechst (blue) and Caspr (red) across vehicle, lysolecithin + vehicle, and lysolecithin + 100 nM PIPE-791. Scale bar 100 µm. Inset is zoomed Caspr staining from vehicle treated sample.

We next used an ex vivo organotypic brain slice culture to assess remyelination following a demyelinating insult. Mouse cortical brain slices were treated with lysolecithin, which induces acute demyelination through a non-specific lipid-based mechanism^38^. Lysolecithin was removed 18 h later and replaced with media containing PIPE-791. After 3 additional days, the brain slices were processed for *Mbp.* We observed a decrease in *Mbp* following lysolecithin insult in vehicle treated slices and a dose-dependent increase in *Mbp* in slices treated with PIPE-791, EC_50_ of 12.1 nM (Fig. 7A).

Slices were also evaluated using a histological endpoint. Here, following demyelination, slices were incubated in PIPE-791 for 5 days, then fixed and processed for immunostaining against MBP. Caspr clustering was used as a marker for nodes of Ranvier formation which implicates the presence of functional axons that have responded to myelination^39,40^. We quantified MBP^+^ area and observed a dose dependent increase following PIPE-791 treatment with an EC_50_ of 74 nM (Fig. 7B). We also observed an increase in the number of Caspr puncta with an EC_50_ of 17.9 nM (Fig. 7C).

Altogether, these data suggest that following a demyelinating insult, PIPE-791 promotes OPC differentiation and induces the formation of functional, myelinating oligodendrocytes in an organotypic slice culture.

### PIPE-791 efficacious in MOG-EAE

We next tested PIPE-791 in the MOG-EAE (myelin oligodendrocyte glycoprotein-induced experimental autoimmune encephalitis) mouse model. This model recapitulates several of the features thought to occur in MS and is a widely used model of inflammatory demyelination.

**Figure 8:**
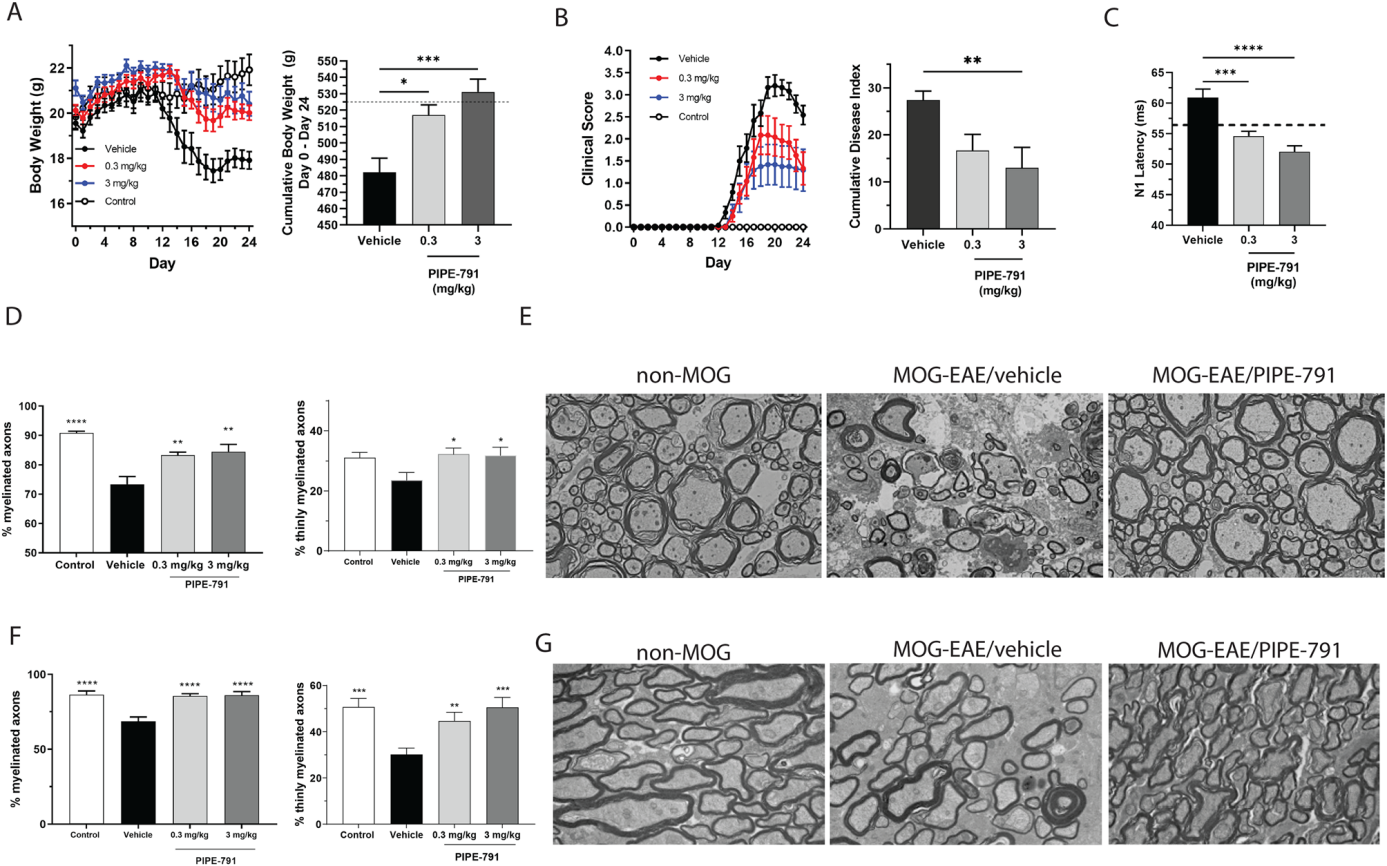
PIPE-791 is efficacious in the MOG-EAE mouse model of multiple sclerosis. (**A**) On Day 0, mice received MOG antigen. Dosing began on day 1 with mice dosed orally, once a day with 0.3 or 3 mg/kg PIPE-791 or vehicle. Left, body weights were measured daily and graphed across time. Error bars are mean ± SEM. Right, Total body weights from day 0 to 24 were summed to generate a cumulative body weight. Significant increases in body weight were observed in both 0.3 and 3 mg/kg PIPE-791 groups (*p = 0.0126, ***p = 0.0003, n = 12, ANOVA with Dunnett’s). (**B**) Left, graph of clinical scores (error bars are mean ± SEM), Right, graph of cumulative disease index (sum of clinical scores from day 0 to 24) with significant improvement observed at 3 mg/kg (mean ± SEM **p = 0.0092, n = 12, ANOVA with Dunnett’s). 0.3 mg/kg had an improved CDI, but did not achieve significance (p = 0.0575, ANOVA with Dunnett’s). (**C**) VEP latencies were measured from non-MOG (dotted line) and MOG-EAE mice treated with vehicle, 0.3 or 3 mg/kg PIPE-791 (***p = 0.0003, ****p < 0.0001, mean ± SEM, n = 12 animals, ANOVA with Tukey’s), (**D**) Left, Graph of myelinated axons in the lumbar spinal cord (g-ratio < 1) of non-MOG mice (****p < 0.0001, n > 8 for all groups) or MOG-EAE mice treated with vehicle, 0.3 mg/kg PIPE-791 (p = 0.0041), or 3 mg/kg PIPE-791 (**p = 0.0013; mean ± SEM). Right, bar graph of % thinly myelinated axons, a surrogate measure of remyelination (1 ≥ g-ratio ≥ 0.8, means ± SEM, *p < 0.05, ANOVA with Dunnett’s, n > 8 for all groups). (**E**) Representative electron micrographs from spinal cord of non-MOG or MOG-EAE with vehicle or PIPE-791 (3 mg/kg). (**F**) Left, graph of myelinated axons in the optic nerve (g-ratio < 1) of non-MOG mice or MOG-EAE mice treated with vehicle, 0.3 mg/kg PIPE-791, or 3 mg/kg PIPE-791 (****p < 0.0001, mean ± SEM, n > 8 for all groups). Right, graph of thinly myelinated axons (1 ≥ g-ratio ≥ 0.8, **p < 0.01, ***p < 0.01, ANOVA with Dunnett’s, n > 8 for all groups). (**G**) Representative electron micrographs of optic nerve from non-MOG or MOG-EAE with vehicle or PIPE-791 (3 mg/kg).

During the EAE model, body weights provide a simple, real-time indication of disease severity. In the vehicle-dosed MOG group, the mean body weight decreased from Day 11 until Day 19 at which time mean body weights were relatively stable until study termination. Although MOG-EAE mice treated with PIPE-791 at 0.3 or 3 mg/kg experienced some weight loss, such loss was significantly mitigated relative to vehicle (Fig. 8A). The mean cumulative body weight of the vehicle group over the duration of the experiment was significantly lower than that of the non-MOG control group and this effect was mitigated in the PIPE-791-treated groups (Fig. 8A).

Clinical disability was also measured as an indicant of disease severity. In the vehicle group, onset occurred at day 13, reaching a mean peak score of approximately 3.2, then decreased to a mean score of 2.5 at the end of the study (Fig. 8B). The mean cumulative disease index (CDI) in the vehicle group was 27.4 by study end. In contrast, when dosed with PIPE-791 at 0.3 or 3 mg/kg, clinical disability onset was delayed, and scores were reduced. Specifically, in mice dosed with 3 mg/kg PIPE-791, disease onset was delayed to day 14 and peak clinical scores only reached 1.4. At study end, scores had reached 1.5. Efficacy was also seen at 0.3 mg/kg, again with delayed onset (to day 14), a peak score of 2.1, and a score of 1.5 by day 24 (Fig. 8B). When clinical scores from day 1 through 24 were summed to generate a cumulative disease index, significant improvement was seen at 3 mg/kg PIPE-791 with trending improvement at 0.3 mg/kg (p = 0.0575, Fig. 8C).

Clinical score is largely influenced by demyelination in the lumbar spinal cord; hence g-ratios (the ratio of the inner to outer diameter of a myelinated axon) were quantified in this area. In control, non-EAE mice, 90.9% of the axons were myelinated (g-ratio < 1). In vehicle treated MOG-EAE mice, only 73.4% of axons were myelinated. When MOG-EAE mice were treated with 3 mg/kg PIPE-791, we observed significant improvement with 84.6% of the axons myelinated. Significant improvement was also seen at 0.3 mg/kg with 83.3% of the axons myelinated.

Remyelinated axons canonically have thin myelin^41^. As such, we examined axons with g-ratios ≥ 0.8 but less than 1 as a surrogate measure of a remyelinating axon. Analysis of these thinly myelinated axons suggest that there was significant remyelination after 3 or 30 mg/kg PIPE-791. Specifically, where MOG-immunized mice showed only 23.6% axons with a 1 > g-ratio ≥ 0.8, with PIPE-791 treatment, this percentage was significantly higher, namely 32.3% at 0.3 mg/kg, and 31.9% at 3 mg/kg. Non-MOG control animals showed 31.2% (Fig. 8D,E).

To further assess myelin status, we measured conductance velocity in the optic nerve, a heavily myelinated tract that is sensitive to demyelination. To measure conductance, we utilized visual evoked potentials (VEPs), a measure for which clinical translatability makes it an especially salient endpoint^42,43^. VEPs were measured on day 21. Non-EAE control mice exhibited an N1 latency of 56.4 ms. In comparison, latency in MOG immunized animals had slowed to 60.9 ms. In mice treated with PIPE-791, significantly shorter latencies were observed, namely 52 ms in the 3 mg/kg, and 54.6 ms in the 0.3 mg/kg groups. Since conduction velocity is driven largely by myelination, these data suggest that more axons were myelinated in the PIPE-791 treated groups (Fig. 8C).

To confirm this, g-ratios in the optic nerve were similarly quantified. Like spinal cord, a loss in myelinated axons was observed in MOG-immunized mice. In non-MOG-EAE mice, 86.4% of the axons were myelinated. MOG-EAE resulted in a reduction to only 68.7% axons being myelinated. With 3 mg/kg PIPE-791, 86.1% of the axons were myelinated. At 0.3 mg/kg, 85.5% were myelinated. When thinly myelinated axons were quantified, 30.2% in the MOG-treated vehicle group were observed, compared to 50.7% in the non-MOG control. Upon treatment with 0.3 mg/kg PIPE-791, this increased to 44.7% and with 3 mg/kg, 50.6% axons were thinly/remyelinated (Fig. 8F,G).

Because our in vitro data suggested that PIPE-791 might inhibit microglial activation, we further performed immunostaining of the spinal cord with an IBA1 antibody. We observed a significant increase in the number of activated microglia in MOG-EAE mice compared to non-MOG-EAE. In mice treated with PIPE-791 (3 mg/kg), the level of IBA^+^ staining was significantly reduced (Supplementary Fig. 9).

## Discussion

Multiple sclerosis is an inflammatory disease that causes profound demyelination, resulting in deficits in signal transduction and subsequent neurodegeneration. Due to its complex etiology, a multifaceted approach to MS, either by way of combination therapies or by the identification of single targets that act on multiple pathways, would be advantageous. With its involvement in OPC differentiation, inflammation, and fibrosis, antagonizing LPA1 presents such a possibility for addressing several of the mechanisms that drive MS. While LPA concentrations are not high in the healthy brain, in diseased contexts such as MS, there is evidence of elevated LPA levels in the CNS, likely due to blood infiltration from the periphery or through increased production in the CNS. In support, we observed the presence of autotaxin expressing HLA-DR^+^ cells proximal to MS lesions. As macrophage incursion is a key feature of MS, we focused our attention on these cells, specifically asking whether macrophage-derived secreted factors could impact OPC differentiation. We show that macrophages secrete diffusible factors (one of which may be LPA) that inhibit OPC differentiation. Direct application of LPA also inhibited OPC differentiation into oligodendrocytes.

We have identified a small molecule antagonist against LPA1, PIPE-791. PIPE-791 is a selective, brain-penetrant, orally bioavailable, small molecule LPA1 antagonist. Due to its binding kinetics, PIPE-791 persists in the brain long after clearance from the plasma. Such extended CNS coverage poses several advantages such as reduced dosing frequency. It is worthwhile highlighting that while most small molecule ligands against GPCRs typically have half-lives on the order of minutes^44,45^, tiotropium (Spiriva) is another rare example of a small molecule GPCR antagonist with slow kinetics, having a t_1/2_ of 24.5 h on the muscarinic M3 acetylcholine receptor^46^.

We have shown that PIPE-791 promotes OPC differentiation into oligodendrocytes and that these oligodendrocytes are capable of wrapping axons in dissociated culture. Mechanistically, LPA signals via LPA1 to a number of pathways including Rho kinase^47^. While further investigation into the signaling mechanisms surrounding LPA1-mediated OPC differentiation are needed, previous work from Pedraza et al.^48^ has shown that inhibition of the Rho pathway with fasudil, an inhibitor of ROCK (Rho-associated kinase) and a known LPA1 effector, leads to OPC differentiation and may be thus be a possible mechanism^48^.

In vivo*,* direct dosing of PIPE-791 in naïve mice induced an increase in CC1^+^ oligodendrocytes relative to vehicle. This suggests that a single dose of PIPE-791 can induce OPC differentiation. It also implies that a baseline level of LPA is present in the brain. Given the dearth of reports on LPA in the naïve brain, further investigation around this observation is warranted. We also showed that PIPE-791 increases the number of CC1^+^ oligodendrocytes in an adult human brain slice culture.

In acutely demyelinated mouse brain slice cultures, following PIPE-791 treatment, we observed an increase in *Mbp* RNA, MBP protein, as well as an increase in the node of Ranvier marker, Caspr. PIPE-791 also reduces both microglial and fibroblast activation in vitro. Next, using the MOG-EAE model of inflammatory demyelination, we showed that PIPE-791 dose-dependently improved functional recovery as measured by both body weight loss and clinical disability score. Furthermore, both 0.3 and 3 mg/kg doses of PIPE-791 resulted in significant improvement in optic nerve myelination as assessed by VEP (a highly sensitive, clinically translatable endpoint). Importantly, we saw significant restoration of myelin at both 0.3 and 3 mg/kg PIPE-791 treatment groups as measured by electron microscopic analysis of g-ratios in both the spinal cord and the optic nerve.

Several LPA1 antagonists such as BMS-986278 or BMS-986020 have demonstrated Phase 1 tolerability and have either progressed to Phase 2 (BMS-986278) or have completed a Phase 2 study (BMS-986020)^49,50^. Current small molecule antagonists are generally intended for peripheral indications such as idiopathic pulmonary fibrosis; thus little is known about LPA1 inhibition in the human CNS. Observations in rodent models may provide possible insight, and although LPA1 null and conditional knockout mice do exist, knockouts with the temporal kinetics of a small molecule, e.g., an inducible knockout, have not been generated. As such, the observations made in constitutive knockouts may not reflect antagonism with a small molecule. Contos et al., observed that LPA1 knockout mice had 50% neonatal lethality and was likely due to developmental abnormalities in the olfactory bulb or cortex^51^. Further, in addition to cells of oligodendroglial lineage, LPA1 is also expressed in neural progenitor cells, particularly in the subventricular zones of mice. Interestingly, while LPA1 labels neural progenitor cells in the mouse, LPA1 is not co-expressed with markers for human neural progenitors. Specifically, LPA1 had been considered a marker for the Gra.Neu5 cluster. Upon closer examination, however, it appeared that the cluster consisted of oligodendrocytes and granule cells and LPA1 had been unintentionally misassigned to the granule cell population^52,53^. Thus, while mouse data implicates a role for LPA1 in neurodevelopment, given the constitutive nature of a knockout and the discrepancies between mouse and human, it remains to be seen how a brain-penetrant, small molecule LPA1 antagonist will behave in human.

In conclusion, these data describe the characterization of PIPE-791, an LPA1 antagonist with favorable CNS drug-like properties. These data also confirm a role for LPA1 in OPC differentiation and remyelination both in vitro and in vivo. Further, we show that antagonizing LPA1 with PIPE-791 may address other mechanisms involved in the manifestation of MS, such as neuroinflammation. Together, these data support LPA1 as a target for remyelination and pave the way for an oral, “first-in-class” LPA1 antagonist for the treatment of MS and other diseases associated with myelin loss.

## Materials and methods

### Animal care and tissue collection

Mice and rats were multi-housed in Innorack IVC mouse racks (Innovive, San Diego, CA) with access to water and standard rodent chow ad libitum. Animals were subject to a 12h light–12h dark cycle. For tissue collection, rodents were euthanized by exposure to CO_2_ followed by cervical dislocation or decapitation unless otherwise specified. All experiments were conducted in accordance with procedures as approved by the Contineum Therapeutics Institutional Animal Care and Use Committee. Reporting of animal studies described in this manuscript followed ARRIVE guidelines.

### Statistics

Unless otherwise noted, statistics were performed using GraphPad Prism software (San Diego, CA) using the specific analyses described in text and legends.

### OPC differentiation assay

*OPC isolation*: For immunopanning purification of OPCs, cortical hemispheres were dissected from postnatal day 8 Sprague–Dawley rat pups. Tissue culture dishes were incubated overnight with goat IgG secondary antibodies to mouse (Jackson Laboratories, Bar Harbor, ME). Dishes were rinsed and incubated at room temperature with primary antibodies for Ran-2, GalC and O4 (gift from Jonah Chan lab). Rodent cortical hemispheres were minced and dissociated with papain (Thermo Fisher, Waltham, MA) for 1 h at 37°. After dissociation, cells were resuspended in a panning buffer (0.2% BSA in DPBS) and incubated at room temperature sequentially on three immunopanning dishes: Ran-2 and GalC were used for negative selection before positive selection with O4.

*OPC Assay:* OPCs were plated in poly-l-lysine (Millipore Sigma, St. Louis, MO) coated 96-well Viewplates™ (Perkin Elmer, Boston, MA) at a seeding density of 35 × 10^5^ cells per well. Cells were maintained in media composed of DMEM (Invitrogen, Carlsbad, CA) supplemented with B27 (Invitrogen, Carlsbad, CA), N2 (Invitrogen, Carlsbad, CA), penicillin–streptomycin (Invitrogen, Carlsbad, CA), N-acetylcysteine (Millipore Sigma, St. Louis, MO), forskolin (Millipore Sigma, St. Louis, MO), and 25 ng/ml PDGF-AA (Peprotech, Cranbury, New Jersey) for 24 h at 37°, 5% CO_2_. Compounds were diluted 10× at a concentration range of 0.001–3 µM. After 24 h, media was gently removed from cells and the same media minus PDGF ± compound was added. Cells were returned to incubator at 37 °C, 5% CO_2_ for 72 h. For immunofluorescence, OPCs were fixed with 4% paraformaldehyde and Hoechst in PBS. Well plates were blocked with 20% normal goat serum and incubated with rat monoclonal antibody to MBP (Millipore Sigma, St. Louis, MO) overnight at 4 °C, and then incubated with secondary antibodies for 1 h at room temperature. For the oligodendrocyte protection assay, 2 h after plating, a mixture of TNFα and IFNγ (both Peprotech, Cranbury, NJ) diluted to a final concentration of 1 ng/ml and 10 U/ml respectively in OPC complete media minus PDGF ± 30 ng/ml T3 or various concentrations of PIPE-791 were added. Cells were returned to incubator at 37 °C, 5% CO_2_.

*LPA1 qPCR:* OPCs were lysed and RNA extracted using the RNEasy Mini kit (Qiagen, Germantown, MD). Reverse transcription was performed using QScript (QuantaBio, Beverly, MA) followed by qPCR with Perfecta PCR SuperMix (QuantaBio, Beverly, MA) on a StepOne Plus Thermocycler. Primers used were as follows: 18S rRNA F: GTCTGTGATGCCCTTAGATG R: AGCTTATGACCCGCACTTAC; mLPA1: F:GGACACCATGATGAGCCTTCTG,R:TCTCATAGGCCAGGACATCGCA; mLPA2 F:TTCTATGTGCGTAGACGGGTGG, R:TGTCCAGCACACCACAAATGCC; mLPA3 F: ACACACCAGTGGCTCCATCAGC, R:GCACGTTACACTGCTTGCAGTTC; mLPA4 F:AGGCTTCTCCAAACGTGTCTGG, R:GGAGGGTTCTAAGCACCACAGA; mLPA5: F:CGTCCCACTGCACGTACAA, R:CAGCAGAAAGCCTAGTATCTCG. Cycling parameters: 95 °C 30 s, then repeat 45×: 95 °C for 5 s, 60 °C for 15 s, followed by melt curve analysis. Data was calculated using 2^−ΔΔCt^, normalizing to 18S RNA and vehicle.

### Human MS brain immunohistochemistry

Formalin fixed, paraffin embedded slides were obtained from Discovery Life Sciences (Huntsville, AL) from a 72 year old male donor with confirmed multiple sclerosis. Tissue from a second donor with confirmed multiple sclerosis was also stained, but information beyond diagnosis of MS was not available (Supplementary Fig. 4). Tissue was deparaffinized in Histoclear (2 × 10 min washes), then rehydrated with an ethanol series (100% 2 × 10 min, 95% 5 min, 70% 5 min, 50% 5 min) then 2 rinses in deionized water. Antigen retrieval was performed at 90 °C for 10 min with 10 mM Tris, 5 mM EDTA, 0.01% Triton X-100, pH 9. Slides were then rinsed 3 times in PBS. Antibodies against autotaxin (goat, 1:250, LSBio, Lynwood, WA), HLA-DR (LN3, mouse, 1:250, ThermoFisher, Waltham, MA), Olig2 (rabbit 1:250, Invitrogen, Carlsbad, CA) were incubated in PBS and 0.1% Triton-X100 overnight. Antibodies were washed off with 3 × 10 m PBS with 0.1% Triton X100. Secondary antibodies were diluted in PBS (all made in donkey, 1:250, α-goat Alexa 647, α-mouse Alexa 546, α-rabbit Alexa 488 from Invitrogen, Carlsbad, CA) and co-incubated with Hoechst (1:2000, Invitrogen, Carlsbad, CA). Slides were washed 3 × 5 m with PBS, mounted and imaged with an Nikon A1R confocal.

### OPC-macrophage Transwell cultures

OPCs were isolated as previously described. Cells were plated on the membrane layer of poly-l-lysine (Millipore Sigma, St. Louis, MO) coated Transwell culture plates (VWR, Radnor, PA) at a seeding density of 1 × 10^6^ cells per well. Transwell culture plates have microporous membrane which allows for factors to flow between the top and bottom chamber. This permits us to minimize direct contact between the macrophage and OPCs while allowing for a constant, renewable source unlike conditioned media which can be depleted. Cells were maintained in media composed of DMEM (Invitrogen, Carlsbad, CA) supplemented with B27 (Invitrogen, Carlsbad, CA), N2 (Invitrogen, Carlsbad, CA), penicillin–streptomycin (Invitrogen, Carlsbad, CA), *N*-acetylcysteine (Millipore Sigma, St. Louis, MO), forskolin (Millipore Sigma, St. Louis, MO), and 25 ng/ml PDGF-AA (Peprotech, Cranbury, New Jersey) for 24 h at 37°, 5% CO_2_. Compounds were diluted 10X at the concentrations reported. After 24 h, media was gently removed from cells and the same media minus PDGF ± compound was added. Cells were returned to incubator at 37 °C, 5% CO_2_ for 1 h.

*Isolation of macrophages.* Male Sprague Dawley rat macrophages were isolated from intraperitoneal lavage fluid. Using a 10 ml syringe, cold PBS −/− was injected into the intraperitoneal cavity. After 30 s with gently agitation, fluid is removed using a transfer pipette. Solution is then spun down at 300g. Pellet is washed once with PBS −/− and allowed to sit for 5 min at room temperature with red cell lysis buffer. Solution was spun again at 300 g and resuspended in DMEM (Invitrogen, Carlsbad, CA) supplemented with B27 (Invitrogen, Carlsbad, CA), N2 (Invitrogen, Carlsbad, CA), penicillin–streptomycin (Invitrogen, Carlsbad, CA), *N*-acetylcysteine (Millipore Sigma, St. Louis, MO) forskolin (Millipore Sigma, St. Louis, MO). Macrophages were plated at 6 × 10^5^ cells per well on the bottom layer of the Transwell plate. Plates were then incubated at 37 °C, 5% CO_2_. *Immunostaining*. After 48 h, the top layer containing the OPC’s were moved to a new plate. Both OPC and macrophage cultures were fixed with 4% PFA plus Hoechst (Thermo Fisher, Waltham, MA), permeabilized and blocked by incubation with 20% goat serum (Millipore Sigma, St. Louis, MO) and 0.1% Triton X-100 (Millipore Sigma, St. Louis, MO) in PBS. Differentiated oligodendrocytes were detected with a rat monoclonal antibody to MBP (Millipore Sigma, St. Louis, MO) and OPCs were detected with a rabbit monoclonal antibody to PDGFRα (Santa Cruz Biotechnology, Dallas, TX). Macrophages were detected with CD68 (Santa Cruz Biotechnology, Dallas, TX). Fluorescent images from cultured oligodendrocytes and macrophages were collected using a Nikon A1R confocal microscope. Images were acquired using a 20× objective, each well was read in a 5 × 5 matrix. Data were analyzed using GraphPad Prism (La Jolla, CA) and statistical significance was determined by ANOVA.

### Determination of LPA concentration in culture media

Rat macrophages were cultured as described above. Media from wells at 30 m or 48 h post-plating were taken. Media from macrophages treated with 1 µM PF-8380 (autotaxin inhibitor, Sigma, St Louis, MO) for 48 h was also collected. For LPA determination, 20 µl of culture media was aliquoted into glass 12 × 75 mm culture test tubes. 500 µl of 30 mM citric acid/40 mM disodium phosphate buffer (pH 4.0) with 1 ng/ml 17:0 LPA as an internal standard (IS) were also added to the test tubes. 2 ml of 1-butanol were then added to the test tubes, tubes covered with parafilm, vortexed for 10 s, and centrifuged for 10 min at 1000*g* at 4 °C. After centrifugation, the top layers were transferred to clean test tubes. The bottom layers were extracted again with 1 ml of water-saturated butanol. The test tubes were covered with parafilm, vortexed for 10 s, and centrifuged for 10 min at 1000*g* at 4 °C. After the second centrifugation step, the top layers were extracted and pooled into the glass tubes containing the first extractions. The pooled extractions were then dried under a nitrogen stream at 25 °C using a nitrogen drier. When the samples were dry, they were reconstituted with 100 µl of methanol, vortexed for 10 s, and transferred to low-volume HPLC vials with 300 µl fused inserts. After reconstitution, the samples were analyzed by LC–MS/MS using an AB Sciex 6500 + .

Chromatographic separation was conducted using a reversed-phase column (Luna Omega C18 1.6 µm, 100 × 2.1 mm). The temperatures of the autosampler and column were set to 15 and 40 °C, respectively. The injection volume was 10 µl. The LC flow rate was set to 0.2 ml/min. Gradient initial conditions were 80% mobile phase A (95:5 water:methanol, 5mM ammonium acetate, 0.1% formic acid) and 20% mobile phase B (5:95 water:methanol, 5 mM ammonium acetate, 0.1% formic acid). After one minute of initial conditions, mobile phase B increased to 85% in 1 min. Mobile phase B then increased to 100% over 7 min. Mobile phase B was held at 100% for 6 min, then decreased to 20% in 1 min. Final conditions were held for another minute before the next injection.

Liquid chromatography was coupled to an AB Sciex 6500 + mass spectrometer. The analysis was conducted under negative ionization mode with turbo ion-spray voltage at − 4500 V, turbo-ion-spray source temperature at 500 °C, and curtain gas at 30 psi. Sample analysis for the analyte, LPA 18:1, was performed using multiple reaction monitoring (MRM). The mass transition used for LPA 18:1 was 435.141 > 153.013. Declustering potential (DP), collision energy (CE), and collision cell exit potential were set at – 95 V, − 30 V, and – 17 V, respectively.

Finally, a qualitative analysis was performed by comparing peak area ratios of analyte/IS among test samples. Citric acid anhydrous (Fisher Chemical Cat# A940-500 lot 231152), Disodium phosphate (Sigma Cat# S9763-100g, lot pcode 1003539106), Test tube (VWR Cat# 47729-570 size 12 × 75 mm), 1-butanol (Sigma Cat# 537993-4L source SHBQ7610), Methanol (Fisher Chemical Cat# A456-500).

### Recombinant membrane binding

*Binding assay:* Membranes were prepared from B103 cells stably expressing human LPA1 receptor. Cells were resuspended in a hypotonic buffer containing 0.25 M sucrose/0.2 mM EDTA. After 30 min on ice, cells were centrifuged at 41,415 × *g*. Membrane pellet was resuspended in 20 mM HEPES, pH 7.4 storage buffer and centrifuged at 41,415 × *g*. Membranes were resuspended in storage buffer + 1 mM dithiothreitol. Protein was quantified using the DC Protein Assay (Bio-Rad, Hercules, CA), aliquoted, and stored at – 80 °C until assay. A tritiated LPA1 selective antagonist^10^ was used as radiolabeled ligand. 3 µl/well of PIPE-791 was serially diluted in 100% DMSO at a concentration range of 0.1 nM to 10 µM; 30 µl/well of radioligand diluted in 50 mM HEPES + 100 mM NaCl, Tween-20 pH 7.4, and 267 µl/well of membrane diluted in 50 mM HEPES + 100 mM NaCl + 2 mM EDTA, pH 7.4. The plates were shaken at 300 rpm for 2 h at room temperature. GF/B plates assay filter plates (Perkin Elmer, Boston, MA, Waltham, MA) were prepared by adding 50 µl of 0.5% polyethylenimine (Millipore Sigma, St. Louis, MO) and incubated at room temperature for > 30 min. The assay was terminated by vacuum filtration (Brandel Harvester). Plates were washed 3 × with 2 ml ice cold buffer containing 50 mM HEPES + 100 mM NaCl + 2 mM EDTA, pH 7.4. Once dry, 50 µl of Betaplate Scintillation Cocktail (Perkin Elmer, Boston, MA) was added to each well. After a 20 min incubation, plates were read on the Perkin Elmer TopCount.

*Kinetics assay:* [^3^H]-PIPE-791 was used as a radioligand. To a deep-well assay plate (Thermo Fisher, Waltham, MA) the following was added: 3 µl/well of either 100% DMSO or 1 µM PIPE-791 diluted in 100% DMSO and 30 µl/well of radioligand diluted in 50 mM HEPES + 100 mM NaCl, Tween-20 pH 7.4 at 0.25, 0.5 and 1 nM. At each time-point 267 µl diluted membranes were added. Fifteen total time points were taken (1 min–24 h). Each time point was assayed in quadruplicate. The plates were placed on a plate shaker at 300 rpm at room temperature. The assay was terminated by using vacuum filtration (Brandel Harvester) to rapidly filter plates through the PEI-coated GF/B plates. The plates were washed 3 × with 2 ml ice cold buffer containing 50 mM HEPES + 100 mM NaCl + 2 mM EDTA, pH 7.4. Once dry, 50 µl of Betaplate Scintillation Cocktail (Perkin Elmer, Boston, MA) was added to each well. After a 20-min incubation to allow the filter to saturate, plates were read on the Perkin Elmer TopCount. All data were analyzed using GraphPad Prism™ software. Dissociation binding was established by measuring the off-rate for [3H]-PIPE-791 dissociating from the receptor using the association kinetics algorithm.

### Functional calcium mobilization assay

B103 cells stably expressing human LPA1 receptor were plated in 96-well black walled, clear bottom plates at 5 × 10^4^ cells/well and incubated for 24 h at 37 °C/5% CO_2_. Intracellular calcium was monitored with Fluo-4 (Molecular Devices). Dye was made up in HBSS with 0.1% (w/v) BSA and 2.5 mM probenecid. Plates were read using a FlexStation 3 (Molecular Devices, Sunnyvale, CA). Calcium response was generated by the simultaneous addition of the EC_80_ of LPA.

*24-h assay:* Antagonists were added to plates prior to cell seeding at a 10× concentration in DMEM (Invitrogen, Carlsbad, CA). On the day of assay, media was removed and replaced with dye and 3× fresh compound in HBSS−/− with 0.1% BSA and incubated for 1 h.

*30-min assay:* Antagonists were added to plates after dye loading was complete at a 3× concentration in HBSS−/− with 0.1% BSA and incubated for 30 min.

*LPA2:* Evaluation of hLPA1 and 2 was conducted at Eurofins (Fremont, CA) using a 3 h preincubation in PIPE-791. Cells were challenged at EC_80_ LPA.

### Cortical myelination assay

Sprague–Dawley rats embryonic day 15 (Charles River) were used. All procedures were approved by the local Institutional Animal Care and Use Committee.

Generation of dissociated culture: Methods used were adapted from those previously described by^22^. Briefly, forebrains were collected from embryonic day 18 rats and finely minced with a scalpel. Tissue was digested in papain (Worthington) for 15 min, washed with 20% HBSS, then triturated with a P1000 pipet. Tissue was centrifuged (500 × *g*), the supernatant removed, the pellet resuspended in Neurobasal (Invitrogen, Carlsbad, CA) supplemented with N21 Max (R&D) and penicillin/streptomycin (Invitrogen, Carlsbad, CA) and plated at a density of 20,000 cells/100 µl/well onto 96-well Viewplates (Perkin Elmer, Boston, MA) coated with poly-d-lysine (Millipore Sigma, St. Louis) and laminin (Millipore Sigma, St. Louis). On day 4, 100 µL of myelination media (MyM) was added. The next day, PIPE-791 was diluted to 3 × of the final concentrations in MyM and 100 µL added to each well.

Immunohistological evaluation of MBP: Cells were fixed 9 days later with 4% paraformaldehyde (EMS) for 15 min followed by three washes with DPBS (Invitrogen, Carlsbad, CA). Cells were incubated in rat MBP (Millipore) and mouse Tuj1 antibodies (BioLegend) diluted in 10% donkey serum containing 0.1% Triton X-100 (Fisher) overnight. Cells were then washed with 3xDPBS followed by 1 h incubation with secondary antibodies (anti-rat, Alexa488; anti-mouse, Alexa647, Hoechst counterstain) in blocking buffer. All secondary antibodies were produced in goat (Invitrogen, Carlsbad, CA) and used at 1:250. Images were acquired using a Nikon A1R confocal microscope and NIS-Elements software. Image analysis was performed using ImageJ. Only MBP segments that co-localized with Tuj1 axons were measured and the average myelin length per oligodendrocyte was calculated.

### Mouse organotypic brain slice culture

Female CD-1 mice at postnatal day 17 (Charles River) were used for the following experiments. All procedures were approved by the local Institutional Animal Care and Use Committee.

Evaluation of *Mbp* transcript in mouse brain slice culture: Brains were collected from P17 mice and placed into ice cold HBSS containing 20% FBS. Brains were bisected sagittally and 250 µm coronal slices made with a manual tissue chopper. Only slices anterior to the hippocampus displaying a discrete corpus callosum were used. Approximately 12 slices are obtained per animal. Slices were laid on a 30 mm MilliCell organotypic culture insert in a 6-well culture dish containing 1.1 mL growth media (DMEM, 25% HBSS-Ca^2+^/-Mg^2+^, 25% heat inactivated horse serum, glucose (5 g/l), 25 mM ascorbic acid and penicillin/streptomycin). Slices were cultured in vitro for 72 h with a 50% media change at 24 h. After 72 h, slices were demyelinated using media containing 0.5 mg/ml lysolecithin for 18 h. Following demyelination, lysolecithin was replaced with media containing varying concentrations of PIPE-791 and treated for 72 h. Slices were snap frozen and RNA extracted using the RNEasy Mini kit (Qiagen). Reverse transcription was performed using QScript (QuantaBio) followed by qPCR with Perfecta PCR SuperMix (QuantaBio) on a StepOne Plus Thermocycler (ABI). Primers used were as follows: 18S rRNA F: GTCTGTGATGCCCTTAGATG R: AGCTTATGACCCGCACTTAC; *Mbp*: CTATAAATCGGCTCACAAGG R: AGGCGGTTATATTAAGAAG. Cycling parameters: 95 °C 30 s, then repeat 45×: 95 °C for 5 s, 60 °C for 15 s, followed by melt curve analysis. Data was calculated using 2^−ΔΔCt^ , normalizing to 18S RNA and vehicle.

Immunohistological evaluation of MBP and Caspr in mouse brain slice culture: Brain slices were cultured as described above. After compound treatment, slices were fixed in 4% PFA for 30 m, blocked in 10% donkey serum, 0.2% Triton X-100, followed by overnight incubation in primary antibodies in blocking solution. Antibodies used were MBP (rat, 1:500, Millipore, Temecula, CA), Caspr (rabbit 1:250 Abcam, Fremont, CA) Tuj1 (mouse, 1:500, Biolegend, San Diego, CA). Slices were washed with 3 × 15 m PBS followed by incubation with secondary antibodies (anti-rat, Alexa488; anti-rabbit, Alexa568, anti-mouse, Alexa647, Hoechst counterstain) in blocking buffer. All secondary antibodies were produced in donkey (Invitrogen, Carlsbad, CA, Carlsbad, CA) and used at 1:250. Filters were excised from insert, mounted with Fluoromount (Millipore Sigma, St. Louis) on a microscope slide and coverslipped. Regions immediately superior to the corpus callosum were acquired using a Nikon A1R confocal microscope and NIS-Elements software. Image analysis was performed using ImageJ. MBP was expressed as MBP surface area normalized to Hoechst cell count; Caspr was expressed as the number of Caspr puncta normalized to Tuj1 intensity.

### Human organotypic slice culture

Human donor brain was received 14 h after death on wet ice. Upon receipt, the brain was immediately placed into-ice cold DPBS without calcium or magnesium. Cortical regions containing gray-white border were isolated then sliced on a McIlwain tissue chopper set at 400 µm thickness. Slices were laid on a 30 mm MilliCell organotypic culture insert in a 6-well culture dish containing plating media (DMEM (Invitrogen, Carlsbad, CA), B-27 (Invitrogen, Carlsbad, CA), 25 mM ascorbic acid (Millipore Sigma, St. Louis), sodium pyruvate (Millipore Sigma, St. Louis), Glutamax (Invitrogen, Carlsbad, CA), and penicillin/streptomycin (Invitrogen, Carlsbad, CA), 1 mM HEPES (Millipore Sigma, St. Louis). Slices were maintained in plating media for a minimum of 1 h, then replaced with culture media. Half of the media was replaced every other day for 10 days. At day 10, culture media was replaced with culture media containing vehicle, PIPE-791 (at 0.3, 3, 30, and 300 nM) or AM152 (at 300 nM) and cultured an additional 9 days, replacing half of the media (containing compound) every other day. Slices were then processed for qPCR or immunohistochemistry.

*Evaluation of Mbp transcript in human brain slice culture:* After compound treatment, slices were immediately transferred to lysis buffer and RNA extracted using the RNEasy Mini kit (Qiagen). Reverse transcription was performed using QScript (QuantaBio) followed by qPCR with Perfecta PCR SuperMix (QuantaBio) on a StepOne Plus Thermocycler (ABI). Primers used were as follows: 18S rRNA F: GTCTGTGATGCCCTTAGATG R: AGCTTATGACCCGCACTTAC; *Mbp*: CTATAAATCGGCTCACAAGG R: AGGCGGTTATATTAAGAAG. Cycling parameters: 95 °C 30 s, then repeat 45×: 95 °C for 5 s, 60 °C for 15 s, followed by melt curve analysis. Data was calculated using 2^−ΔΔCt^ , normalizing to 18S RNA and vehicle.

*Immunohistological evaluation of oligodendrocytes in human brain slice culture:* After compound treatment, slices were fixed in 4% PFA overnight. Slices were gently lifted from the membrane insert and washed with at least five 15 min washes in DPBS containing 0.5% Triton-X 100 (0.5%-DPBS) followed by overnight incubation in primary antibodies in blocking solution. Antibodies used were CC-1 (mouse, 1:250, Millipore, Temecula, CA), Olig2 (rabbit 1:250 CellMarque). Slices were washed with at least five 15 min washes in 0.5%-DPBS followed by incubation with secondary antibodies (anti-mouse Alexa488; anti-rabbit, Alexa568, and Hoechst counterstain) in 0.5%-DPBS. All secondary antibodies were produced in goat (Invitrogen, Carlsbad, CA, Carlsbad, CA) and used at 1:250. Filters were excised from insert, mounted with Fluoromount (Millipore Sigma, St. Louis) on a microscope slide and coverslipped. 2 images per slice were acquired using a Nikon A1R confocal microscope and NIS-Elements software. Images were thresholded and counted using ImageJ.

### Microglial activation and phagocytosis assays

Brains were harvested from mice (postnatal day 20, Charles River). Hippocampi were dissected out and immediately placed into ice cold HBSS containing either 0.1% DMSO or 3 µM PIPE-791 (dissection media). 400 µm thick coronal slices were cut on a McIlwain tissue chopper and returned to dissection media and separated manually. Slices were then transferred into culture media containing DMEM + 0.01% fatty acid free BSA (Gibco) with 3 µM PIPE-791 or DMSO. Slices were incubated for 2 h at 37 °C (4 slices per condition). Microglial activation was initiated by adding LPA (18:1, Avanti Polar Lipids) to a final concentration of 10 µM and incubated for 3 h. Slices were then fixed in formalin and for 2 h.

For immunohistochemistry, slices were washed for 3 × 15 min in 0.5% Triton-X100 and DPBS. Slices were then stained using IBA-1 antibody (Thermo Scientific) in 10% normal goat serum with 0.5% Triton-X100 in DPBS overnight on a rocker. Slices were then washed in 0.5% Triton-X100 in DPBS 3 × 15 min. Goat anti-mouse Alexa 564 (1:500, Invitrogen, Carlsbad, CA) and Hoechst 333421 (Invitrogen, Carlsbad, CA) were diluted in DPBS with 0.5% Triton-X100 and incubated for 2h. Slices were washed 3 × 15 min in DPBS with 0.5% Triton-X100, mounted with Fluoromount and imaged on a Nikon A1R confocal microscope.

For phagocytosis, microglia were harvested from cortical cultures generated from P2 rats. Cortices were isolated, meninges removed, minced and digested in 0.05% trypsin (Invitrogen, Carlsbad, CA, Carlsbad, CA) for 15 min at 37 °C. Tissue was allowed to settle, trypsin removed and HBSS (Corning, Glendale, AZ) with 20% FBS (20% HBSS) was added to halt further digestion. Tissue was washed again in 20% HBSS and resuspended in DMEM with 10% FBS and penicillin/streptomycin (10% DMEM). Cells were plated in a T150 flask and cultured for 10 days with media changes every other day. Microglia were mechanically isolated by tapping flask against the hood surface. Microglia were transferred to a conical and centrifuged at 800 × *g* for 15 min and resuspended in 10% DMEM, plated in 96 wells at a density of 5000/well and cultured for 3 days. Culture media was replaced with fresh 10% DMEM containing PIPE-791 and incubated for 6 h. Fluoromyelin labeled myelin debris was added to each well and incubated for 90 min. Microglia were then fixed and immunostained for IBA1 (Invitrogen, Carlsbad, CA, Carlsbad, CA). Fluoromyelin debris labeling: Myelin was isolated from corpus callosum of adult CD-1 mice and triturated several times through a 25-gauge syringe needle until homogeneous then centrifuged at 19,000 × *g* for 15 min. Supernatant was removed and washed 3 × in PBS then frozen at – 20 °C. Prior to applying to microglia, the pellet was resuspended in PBS, fluoromyelin (Invitrogen, Carlsbad, CA, Carlsbad, CA) was added at 1:300 and incubated for 30m. Labeled myelin was pelleted (19,000 g × 5 m), supernatant removed and resuspended in 10% DMEM.

### Rat meningeal fibroblasts

Meningeal fibroblasts were isolated from postnatal day 8 CD-1 rats (Charles River Labs, Willington, MA). Meninges were peeled off and digested in Accutase (Thermo Fisher, Waltham, MA, Waltham, MA) for 20 min at room temperature. 20% HBSS was added to stop protease activity. Digested meninges were dissociated by trituration and cells were strained through a 40 µm nylon mesh and spun down at 800 g for 15 min. The cell pellet was resuspended in DMEM + 10% FBS and plated in a T-75 flask (Corning) for 10 days. Cells were lifted with Accutase and replated into 96-well Perkin Elmer, Boston, MA Viewplates, at 15k per well. The next day, cells were starved in DMEM for 8 h. PIPE-791 at different concentrations was added and incubated for 15 h. 1 µM LPA or vehicle were added for 5 h. Cells were fixed with formalin, permeabilized with 0.1% Triton X100 in PBS, and incubated with Col1 antibody (1:250, Thermo Fisher, Waltham, MA, Waltham, MA) overnight. Primary antibody was detected using a goat-anti-mouse Alexa488 secondary antibody (Invitrogen, Carlsbad, CA, 1:250) with Hoechst. Wells were imaged on Nikon A1R confocal and Col1^+^ area measured normalized to cell number (Hoechst count).

### Human meningeal fibroblasts

Meningeal fibroblasts were obtained from IxCells (San Diego, CA) and plated at 5000 cells per well according to manufacturer's protocol. After 1 day, cells were starved overnight in DMEM and treated with PIPE-791 for 6 h followed by 4 h with 10 µM LPA. Cells were stained and imaged as described with rat meningeal fibroblasts.

### In vivo dose-occupancy

*Occupancy using [3H]-OPC3497 as radiotracer:* Female C57BL6/N mice (Envigo, Indianapolis, IN) were dosed with vehicle or PIPE-791 by oral gavage. [^3^H]-OPC3497 was diluted to 9.8 µCi/mL in saline and administered via IV injection to the lateral tail vein at a dose volume of 5 ml/kg at the times indicated. Mice were euthanized by decapitation, trunk blood collected into K_3_EDTA tubes and stored on wet ice, each brain rapidly dissected and the forebrain isolated, weighed and placed into a 5 ml polypropylene tube. Tissues were diluted with a 10 × volume of ice-cold binding buffer (50 mM HEPES, 100 mM NaCl, 2 mM EDTA, pH 7.4). Brains were homogenized and 350 µl of the homogenate was filtered over Whatman GF/B filters (GE Life Sciences, Marlborough, MA) pre-wetted with 0.5% polyethyleneimine. Filters were washed with cold 50 mM TRIS–HCl, 154 mM NaCl, 0.05% Tween 20, pH 7.4 twice and dried in an oven (~ 40–50 °C). 5 ml Ultima Gold F scintillation fluid (Perkin Elmer, Boston, MA, Boston, MA) was added to each tube and samples read on a Beckman LS6500 liquid scintillation counter. After all samples were collected, dissected, and filtered, the previously collected blood in K_3_EDTA tubes was centrifuged (1450 × *g*, 10 min, 4 °C) to separate plasma. Plasma was aliquoted into 96-well polypropylene plates and stored at – 80 °C until analysis for PIPE-791 by LC–MS/MS.

*Occupancy using [3H]-OPC3497 as radiotracer:* [^3^H]-PIPE-791 was diluted to 9.8 µCi/ml in saline and administered via IV injection to the lateral tail vein at a dose volume of 5 ml/kg at various times (1 min, 5 min, 30 min, 2 h, 4 h, 24 h, 72 h, 7 days, 10 days, or 14 days) prior to sample collection at Time 0.

A subset of mice from the 2 h and 24 h [^3^H]-PIPE-791 groups were dosed with PIPE-791 (3 mg/kg for non-specific binding) by oral gavage 2 h prior to [^3^H]-PIPE-791 administration. At time 0, mice were euthanized, trunk blood collected into K_3_EDTA tubes and stored on wet ice (PIPE-791-dosed animals only), forebrain isolated, weighed and placed into a 5 ml polypropylene tube. Tissues were diluted with 10 × volume of ice-cold binding buffer. Brains were then rapidly homogenized and 350 µl of the resulting homogenate was filtered in duplicate over Whatman GF/B filters (GE Life Sciences, Marlborough, MA) which had been pre-wetted with 0.5% polyethyleneimine prior to loading onto the Hoefer vacuum manifold. Filters were washed twice by applying 5 ml ice-cold wash buffer to the manifold.

### In vivo CC1 induction assay

Female C57Bl/6 mice (9–11 weeks old) were used in this experiment, n = 5/group. On day 0, mice were dosed with vehicle (1% HPMC, 0.1% Tween-80) or 3 mg/kg PIPE-791. On day 5, mice were sacrificed and brains submerged in formalin. Tissue was sectioned at 40 µm on a sliding microtome (ThermoFisher, Microm HM450, Waltham, MA). Four sections per mouse were immunostained. Sections were permeabilized and blocked by incubation with 20% goat serum (Millipore Sigma, St. Louis, MO) and 0.2% Triton X-100 (Millipore Sigma, St. Louis, MO) in PBS. Oligodendrocytes were detected with a rat monoclonal antibody to Anti-APC (1:500, Calbiochem, San Diego, CA), and the total pool OPC/oligodendrocyte pool were detected with a rabbit monoclonal antibody to Olig-2 (1:500, Invitrogen, Carlsbad, CA). Alexa Fluor 594 and 647 IgG secondary antibodies against rat and rabbit (1:1000, Invitrogen, Carlsbad, CA) were used for primary antibody detection. Cell nuclei were identified with Hoechst (1:1000, Invitrogen, Carlsbad, CA). Fluorescent images from 4 brain slices per mouse with 2 regions of interest (adjacent and superior to the corpus callosum) per brain slice were collected on a Nikon A1R confocal microscope. Detection and quantification of the cells were performed using NIS Elements imaging software (Nikon, Minato, Tokyo).

### MOG-EAE induction and assessment

On Day 0, EAE was induced using a MOG_35–55_/CFA Emulsion PTX Hooke Kit™ (Hooke Laboratories, Lawrence, MA). Briefly, per manufacturer’s instructions, each mouse was administered a total of 0.2 ml emulsion delivered via dorsal subcutaneous injections of 0.1 ml each to the mid-scapula and lower lumbar regions. Pertussis toxin was prepared per lot-specific instructions and administered via intraperitoneal injection of 0.1 ml (200 ng) on Day 0, 2 h post-MOG administration, and again on Day 1, 24 h post-MOG.

Mice were dosed by once daily oral gavage for 25 days with 1% HPMC/0.1% Tween-80 vehicle, 0.3 mg/kg PIPE-791, or 3 mg/kg PIPE-791. Each treatment group contained 12 mice, except for the age-matched control group which contained 8 mice.

Body weights and clinical scores were recorded daily. Clinical scores were assessed as follows:

**Table:** 

Score	Observation
0	Normal
0.5	Limp distal tail
1	Limp/weak tail
1.5	Loss of righting reflex when placed in the prone position
2	Weak hind limbs, waddling gait, legs held close together when picked up, unilateral hind limb dragging
2.5	Bilateral hind limb paresis, dragging both hind limbs or tripping on hind feet
3	Unilateral hind limb or unilateral front limb paralysis
3.5	Bilateral hind limb paralysis, flat hindquarters
4	Mild fore limb paresis or partial front limb paralysis
4.5	Severe fore limb paresis, not alert
5	Moribund or complete bilateral fore and hind limb paralysis

Supportive care (subcutaneous saline, Nutrical supplement, HydroGel) were provided to all animals with a clinical score greater than 3 and/or displaying a body weight loss greater than 10% from the previous day’s weight.

### Visual evoked potentials

VEP recordings were conducted 21–24 days post-MOG induction (for simplification, this will be referred to as a nominal Day 21 VEP throughout this document). To do this, animals were placed in a room with red light conditions and allowed to adapt for 1 h. Animals were anesthetized with an intraperitoneal injection of ketamine/Xyalazine (75/10 mg/kg). Pupils were dilated with 1.0% tropicamide (1 drop per eye) (Akorn Pharmaceuticals, Lake Forest, IL). One minute following application tropicamide, one drop of Genteal (Alcon Laboratories, Fort Worth, TX) was applied to each eye to maintain ocular moisture during anesthesia.

VEPs were conducted using a Celeris system (Diagnosys, Lowell, MA). Anesthetized mice were placed onto a heated platform (37C) and instrumented with subcutaneous recording electrodes placed into the snout and dorsal occipital regions. VEP execution started approximately 10–12 min following injection of the anesthetic. Each examination was comprised of at least 3 runs using settings outline in Table 3. Flash VEPs were recorded from each eye independently and simultaneously. Parameters used (setting): Pulse intensity (3 cd s/m^2^); Frequency (1 Hz); On time (4 ms); Pulse color (white-6500K); Sweeps per result (100). VEP waveforms were analyzed using Espion (Diagnosys, Lowell, MA). N1 latency for each eye was determined by averaging N1 from 3 independent VEP traces. Similarly, P1–N1 and N1–P1 amplitudes were determined. Waveform symmetry and integrity was expressed by calculating the amplitude ratio (AR): AR = (P1–N1 amplitude in µV)/(N1–P2 amplitude in µV). An AR approximately ≥ 1 suggests normal VEP integrity, while an AR < 1 and approaching 0 suggests degradation of the N1-P2 amplitude.

### Electron microscopy and g-ratios

Study termination was the last day that clinical scores were determined for all study animals (Day 24). Since VEP recordings required several days (Days 21–24), oral dosing continued through perfusion, which was performed on Day 24 or Day 25. On Day 24 or 25, and following completion of VEP recordings, mice were deeply anesthetized with isoflurane anesthesia and whole body perfused with EM grade Karnovsky’s fixative (3% glutaraldehyde, 2% paraformaldehyde in 0.1 M phosphate buffer, pH 7.4; part # 15732-10; Electron Microscopy Sciences, Hatfield, PA). Spinal columns and optic nerves were collected and stored in fixative at 4 °C until further processing. After at 3–5 days in fixative, spinal cords were isolated and dissected from the vertebrae. Approximately 2 mm of the most caudal lumbar region was trimmed and segmented and, along with the optic nerves, shipped to Charles River Laboratories (Durham, NC) for processing for and imaging by electron microscopy.

At Charles River Laboratories, tissues (spinal cord or optic nerves) were post-fixed in osmium tetroxide, rinsed in distilled water (2×), dehydrated through an ethanol series (50%, 70%, 95%, 100% (3×)), transitioned through propylene oxide (2x), infiltrated in Epon-Araldite (1:1 EA:PO, 3:1 EA:PO, pure EA), and embedded in Epon-Araldite blocks. The blocks were polymerized overnight at ~ 85 °C, and semi-thin sections (~ 1 μm, one per tissue) prepared and stained with toluidine blue using standard protocols. Semi-thin images were evaluated, and the most caudal region was identified as the region of focus for thin sections. Thin sections of ~ 100 nm were prepared using standard protocols and the grids were imaged at 1500 × on a JEOL JEM-1400 + transmission electron microscope fitted with an AMT 16 MP digital camera system. The direct magnification used was 1500 × or 4000 × for spinal cord and optic nerve, respectively.

Digital EM images were analyzed using ImageJ (NIH, Bethesda, MD) measure the circumference of the axon and, when present, the myelin sheath, and g-ratios were calculated. For spinal cord analysis, 4 representative images were analyzed per animal and the results pooled, and the percentages calculated from the animal total. For optic nerve analysis, 1 representative image was analyzed per optic nerve and each optic nerve was treated as a separate data point.

### Immunohistochemical analysis

A lower lumbar segment of spinal cord was transferred from the EM fixative to 30% sucrose overnight and then sectioned using a sliding microtome. The resulting sections were stored in 30% sucrose in 0.1 M Phosphate Buffer pH 7.4 and stored at 4 °C. Free-floating spinal cord sections were rinsed with PBS, blocked with 5% normal goat serum in 0.1% Triton-X/PBS for 2 h, and subsequently incubated with antibodies against IBA1 (Life Technologies, Carlsbad, CA, 1:500) or CD3 (Abcam, Waltham, MA, 1:500) overnight at 4 °C. Sections were then washed 3 times with 0.1% triton-X/PBS and transferred to secondary antibody (1:500, Carlsbad, CA) in 0.1% Triton-X/PBS for 2 h at room temperature. Hoechst 33342 was diluted 1:2000 and added during the final wash step. Sections were stained with 1% Sudan Black in 70% ethanol, and washed 3 × 5 min in PBS. Stained sections were mounted onto slides and evaluated using a Nikon A1R confocal microscope and analyzed using Nikon NIS Elements software. Regions of interest (ROIs) were selected based on the largest contiguous lesion defined by Hoechst and Sudan Black co-staining. Intensity thresholds were made for each stain to determine IBA1 area. Cells were counted using the automatic General Analysis function in NIS Elements.

### Supplementary Information

Supplementary Information.

## Data Availability

The datasets used and/or analyzed during the current study are available from the corresponding author on reasonable request.
