# Supplementary material for: Discovery of a brain penetrant small molecule antagonist targeting LPA1 receptors to reduce neuroinflammation and promote remyelination in multiple sclerosis
Source: Sci Rep. 2024 May 8;14:10573. doi: 10.1038/s41598-024-61369-9 (PMC11079064; doi:10.1038/s41598-024-61369-9)
Supplement: Supplementary file 1 — Supplementary Information. [file 41598_2024_61369_MOESM1_ESM.docx]

**Supplemental Figure 1**


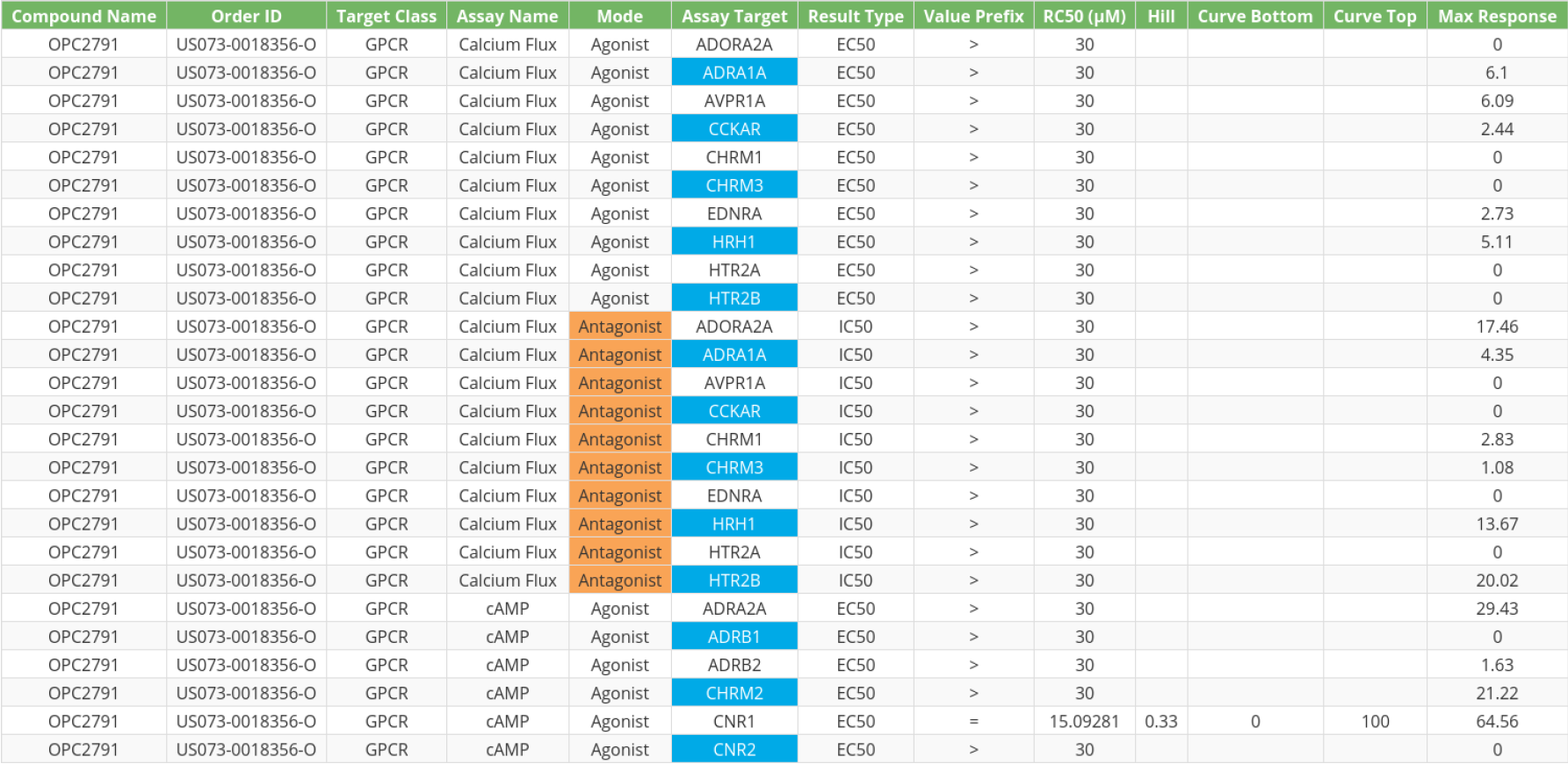


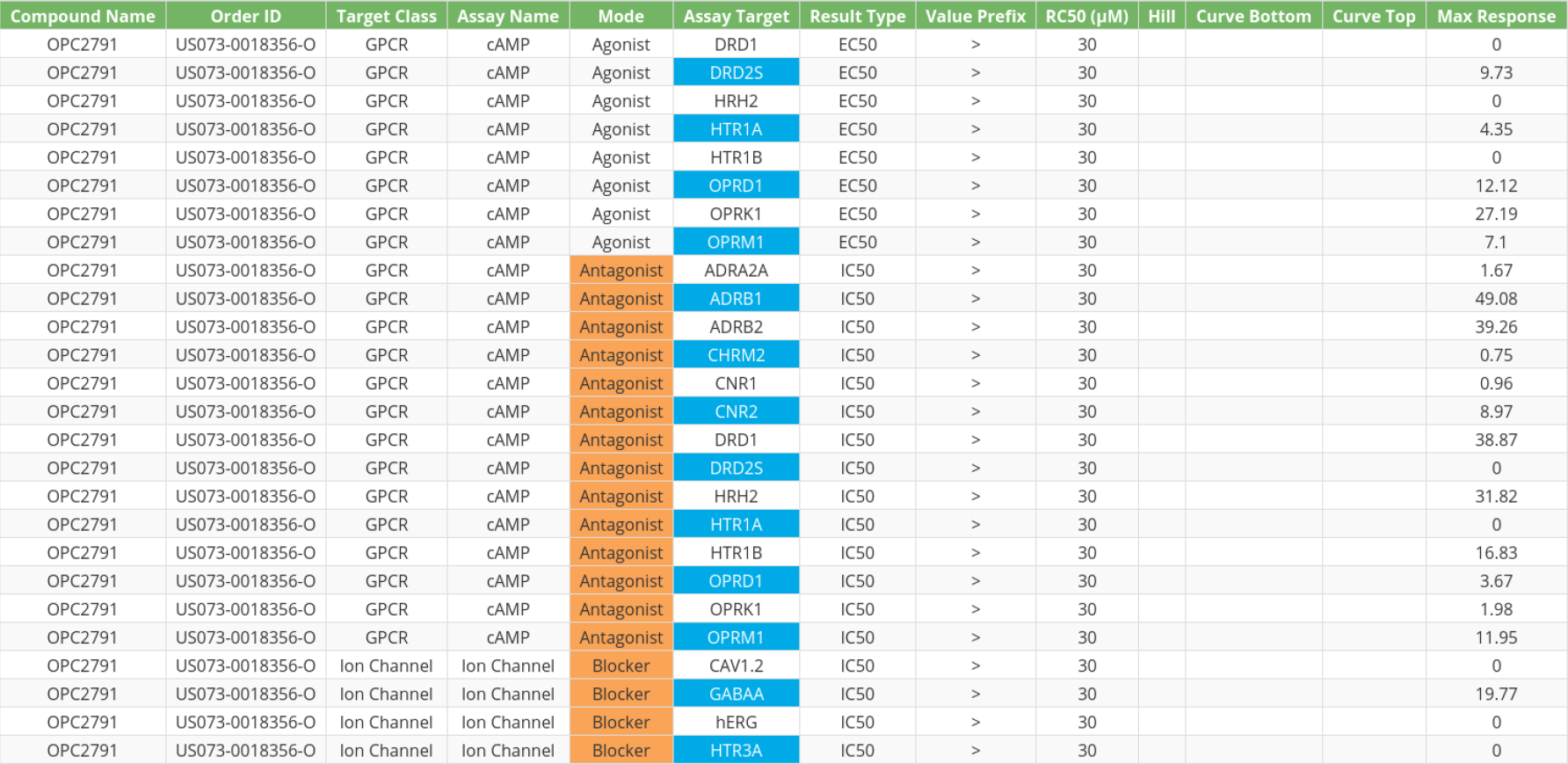


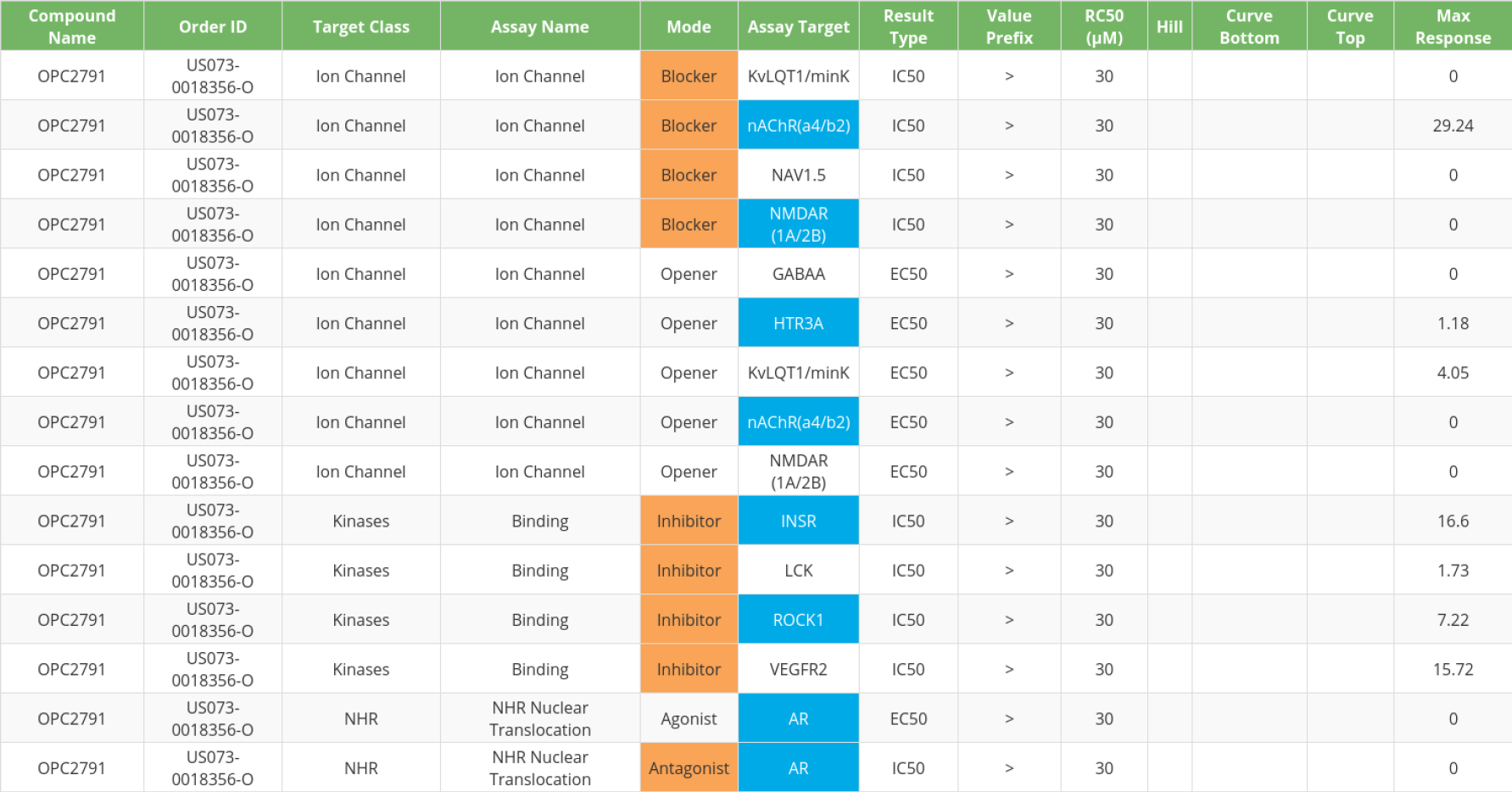


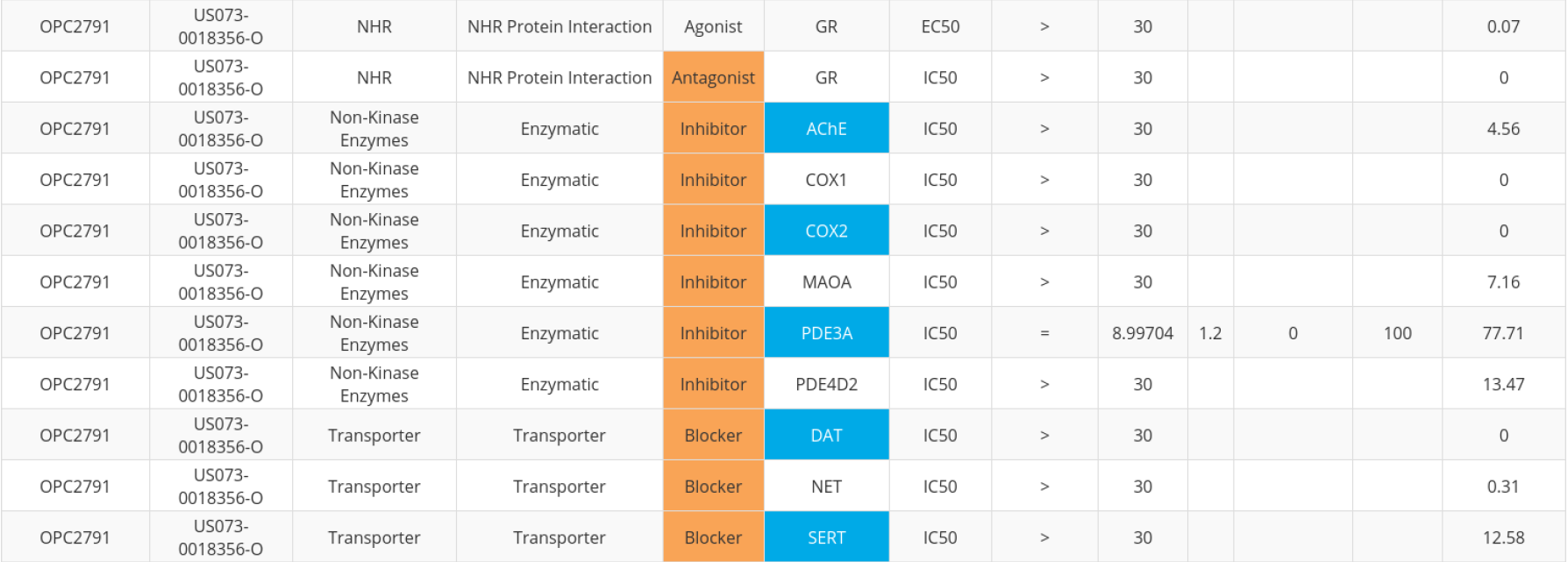


**Supplemental Figure 1.** Results from Eurofins SAFETYscan E/IC50 ELECT. PIPE-791 was tested at a concentration of 30 µM.

**Supplementary Figure 2**

**Supplementary Figure 2.** PIPE-791 has slow on/off association kinetics *in* vivo. Mice were dosed intravenously with 49µCi/kg [^3^H]-PIPE-791 (9.8 µCi/mL at a dose volume of 5 mL/kg) and brains collected at time points listed (black bars). Dashed bars denote timepoints collected at which 3 mg/kg PIPE-791 was orally dosed 2 hours prior to [^3^H]-PIPE-791 injection to assess specific binding (mean ± SEM, n=6).

**Supplemental Figure 3**

**Supplemental Figure 3.** OPC3497 is a suitable radioligand for evaluating LPA1 receptor occupancy. Following IV administration, [^3^H]-OPC3497 binding in mouse brain increased rapidly, peaked 5 minutes post-dose and declined sharply over the evaluation period (2 h). Total binding at 5 minutes and 30 minutes was significantly reduced by PIPE-791 (dosed orally 2 h prior), indicating that [^3^H]-OPC3497 bound to LPA1 *in vivo* with high specific binding and low non-specific binding (mean ± SEM, n=4).

**Supplemental Figure 4**

**
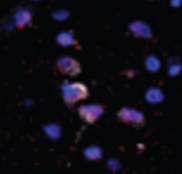
_
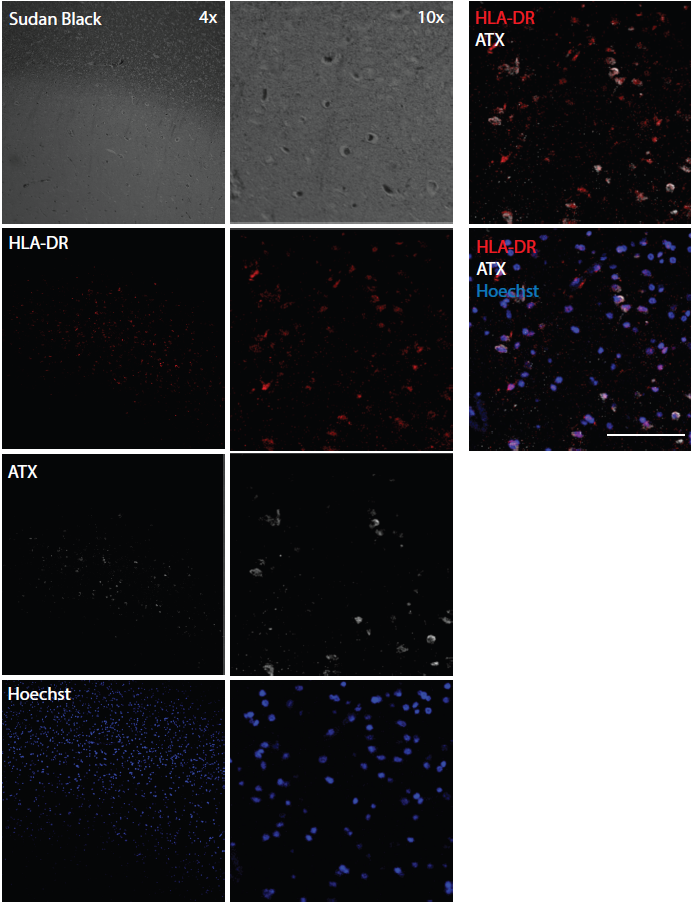
_**

**Supplementary Figure 4.** Tissue from a second MS patient donor was stained with antibodies against HLA-DR (red), autotaxin (white) and counterstained with Hoechst (blue) and the myelin dye, Sudan Black. Myelin containing areas stain dark, while lesion stains lighter. Left column is section taken at 4x. Middle column is at 10x magnification. Yellow dashed box denotes where 10x image was taken. Right column shows co-localization between HLA-DR and autotaxin (top) or HLA-DR, autotaxin, and Hoechst positive cells (middle), magnified image (bottom). Yellow arrowhead denotes cluster of HLA-DR/autotaxin/Hoechst^+^ cells. Image magnified in Scale bar: 100 µm.

**Supplemental Figure 5**

**A B**


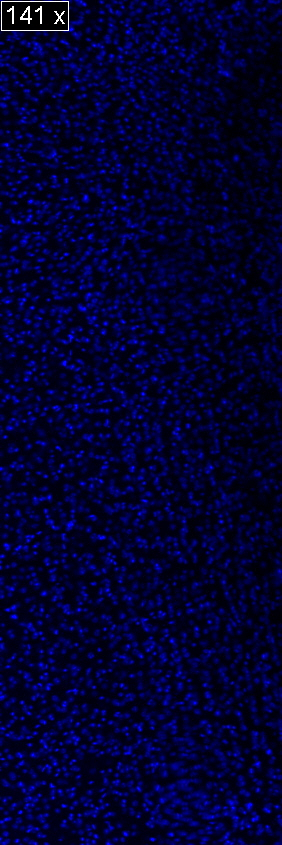

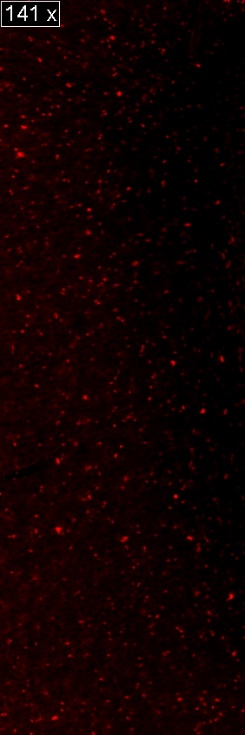

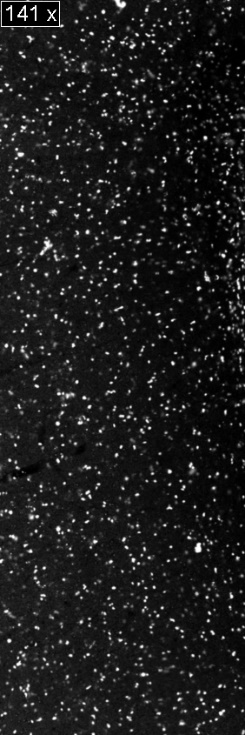

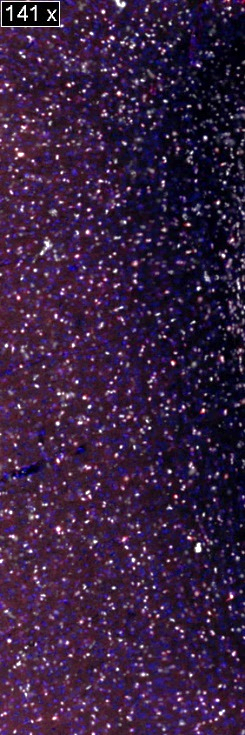


CC1

Olig2

Hoechst

merge

Vehicle


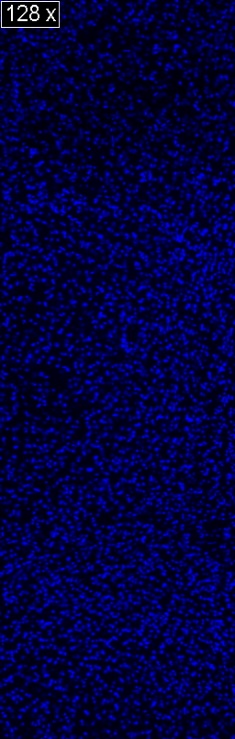

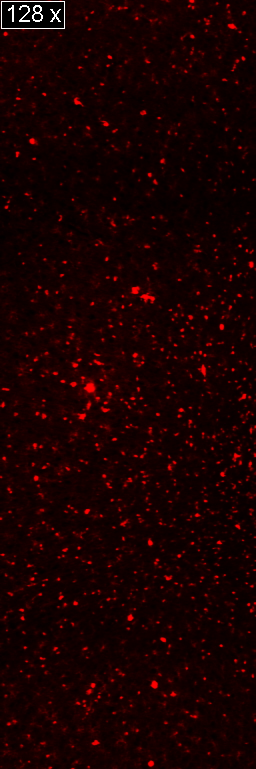

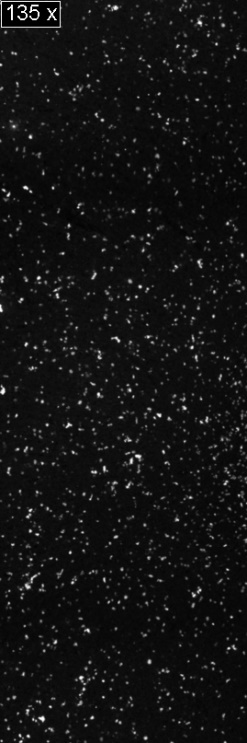

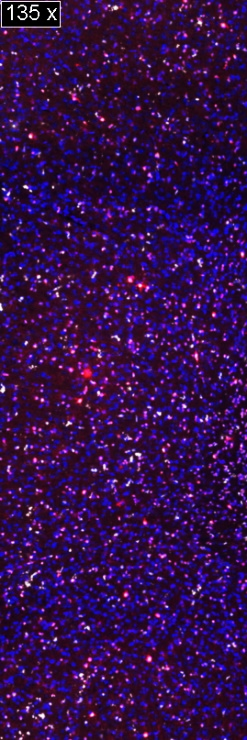


Olig2

merge

PIPE-791

CC1

Hoechst

**Supplemental Figure 5.** PIPE-791 induces OPC differentiation *in vivo*. Mice were dosed once with PIPE-791 (3 mg/kg, PO) and brain tissue collected 5 days later. Brain sections from the same region (cortical region superior and adjacent to the corpus callosum) were collected and imaged. Sections were immunostained using antibodies against CC1 (mature oligodendrocytes), Olig2 (oligodendroglial marker), and counterstained with Hoechst. A significant increase in CC1^+^/Olig2^+^ oligodendrocytes was observed (mean ± SEM, t-test, n=5). B. Representative images of data graphed on A. Sections stained for CC1 (red), Olig2 (white), and counterstained with Hoechst (blue). Scale bar 50 µm.

**Supplemental Figure 6**

**Supplementary Figure 6.** AM152 (LPA1 antagonist) inhibits microglial activation. Mouse hippocampal slices (postnatal day 21) were generated and treated with PIPE-791. LPA was then added to the slices to induce microglial activation. Slices were fixed and stained with an antibody against IBA1 and counterstained with Hoechst. Activation was quantified using cell perimeter of IBA1^+^/Hoechst^+^ cells (mean ± SEM, n=4 slices, ANOVA with Tukey’s).

**Supplemental Figure 7**

**A**

**B**


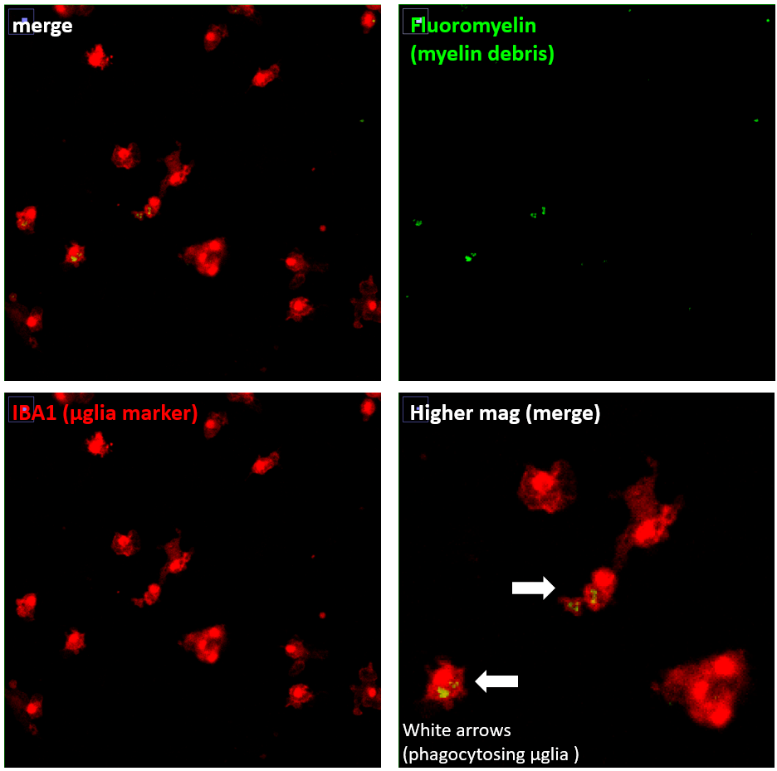


**Supplemental Figure 7.** PIPE-791 does not inhibit myelin debris phagocytosis by microglia. A. Activated microglia were plated and treated with varying concentrations of PIPE-791 for 3 h. Fluoromyelin labeled myelin debris was added and incubated for 90 min. Cells were fixed and immunostained with an antibody against the microglial marker IBA1. Total IBA1^+^ and IBA1^+^/fluoromyelin^+^ cells were counted. Data point represents an image, each color represents a separate well. No significant decrease in engulfment was observed (mean ± SEM, n=4). B. Representative image of fluoromyelin engulfment. IBA (red), fluoromyelin (green). White arrows denote phagocytosing microglia. Scale bar: 25µm.

**Supplemental Figure 8**

1. B.

C.


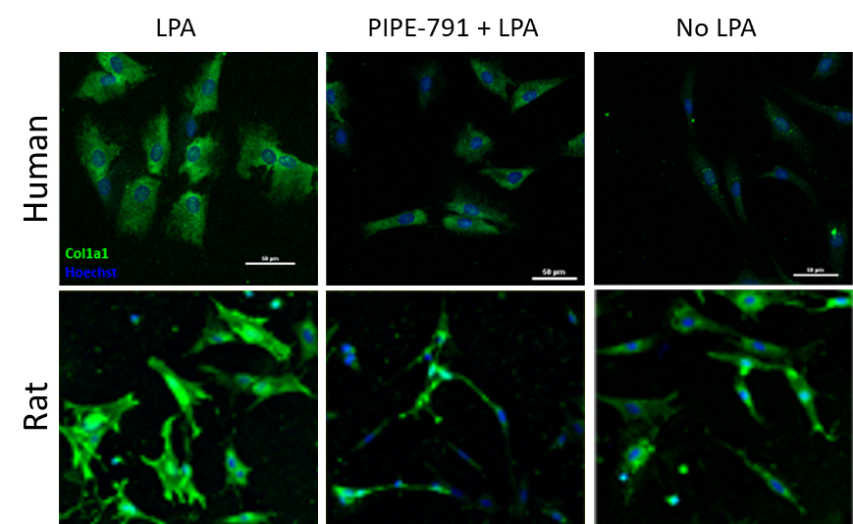


**Supplemental Figure 8.** PIPE-791 prevents LPA induced fibroblast activation. A. Rat primary meningeal fibroblasts were isolated and serum starved overnight. Fibroblasts were pretreated with PIPE-791 for overnight then stimulated with LPA for 5 hours. Cells were fixed and immunostained with a collagen 1a1 antibody (green) and counterstained with Hoechst (blue). Collagen I area was measured with PIPE-791 showing inhibition with an IC_50_ of 31.8 nM (mean ± SD, n=4, AM152 was p=0.003). B. Human primary meningeal fibroblasts were serum starved overnight and pretreated 4h with PIPE-791 then stimulated with LPA for 4 hours. Cells were fixed and immunostained with a collagen I antibody (green) and counterstained with Hoechst (blue). Collagen 1A1 area was measured with PIPE-791 showing inhibition with an IC_50_ of 4.5 nM (mean ± SD, n=4, AM152 was p=0.0365). C. Representative images of human and rat fibroblasts after activation with LPA as described in A and B. PIPE-791 concentration was 1 µM. Collagen 1a1 (green), Hoechst (blue). Scale bar 50 µm. One-way ANOVA with Dunnett’s; * p<0.05, ** p<0.01, *** p<0.001, **** p<0.0001.

**Supplemental Figure 9**


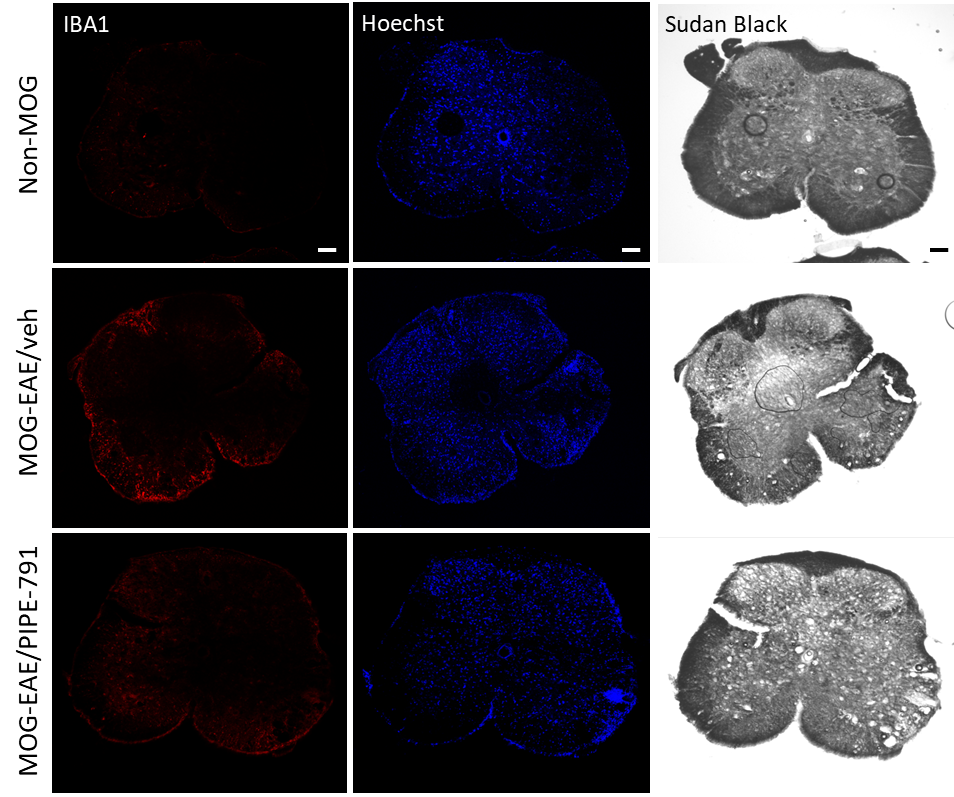

**Supplemental Figure 9**. PIPE-791 reduces IBA1^+^ area in MOG-EAE spinal cord. Sections from MOG-EAE spinal cords treated with vehicle or with 3 mg/kg PIPE-791 were immunostained with an IBA1 antibody and counterstained with Hoechst. Left, IBA1^+^ area was quantified and normalized to Hoechst counts (* p = 0.0166, *** p=0.0002, mean ± SEM, ANOVA with Tukey’s, n>4). Right, representative images of data taken from non-MOG, MOG-EAE treated with vehicle or 3 mg/kg PIPE-791. IBA1 (red) counterstained with Hoechst (blue). Sudan Black was used to visualize the section. Scale bar 100 µm.
